# Fibulin-2 is an extracellular matrix inhibitor of oligodendrocytes relevant to multiple sclerosis

**DOI:** 10.1172/JCI176910

**Published:** 2024-05-14

**Authors:** Samira Ghorbani, Cenxiao Li, Brian M. Lozinski, Dorsa Moezzi, Charlotte D’Mello, Yifei Dong, Frank Visser, Hongmin Li, Claudia Silva, Mohammadparsa Khakpour, Colin J. Murray, Marie-Ève Tremblay, Mengzhou Xue, V. Wee Yong

**Affiliations:** 1 Hotchkiss Brain Institute and Department of Clinical Neurosciences, University of Calgary, Calgary, Alberta, Canada.; 2 Department of Biology, University of Toronto Mississauga, Mississauga, Ontario, Canada.; 3 Department of Immunology, University of Toronto, Toronto, Ontario, Canada.; 4 Department of Biochemistry, Microbiology, and Immunology, College of Medicine, University of Saskatchewan, Saskatoon, Saskatchewan, Canada.; 5 Department of Cerebrovascular Diseases, The Second Affiliated Hospital of Zhengzhou University, Zhengzhou, China.; 6 Division of Medical Sciences, University of Victoria, Victoria, British Columbia, Canada.

**Keywords:** Neuroscience, Extracellular matrix, Multiple sclerosis

## Abstract

Impairment of oligodendrocytes and myelin contributes to neurological disorders including multiple sclerosis (MS), stroke, and Alzheimer’s disease. Regeneration of myelin (remyelination) decreases the vulnerability of demyelinated axons, but this repair process commonly fails with disease progression. A contributor to inefficient remyelination is the altered extracellular matrix (ECM) in lesions, which remains to be better defined. We have identified fibulin-2 (FBLN2) as a highly upregulated ECM component in lesions of MS and stroke and in proteome databases of Alzheimer’s disease and traumatic brain injury. Focusing on MS, the inhibitory role of FBLN2 was suggested in the experimental autoimmune encephalomyelitis (EAE) model, in which genetic FBLN2 deficiency improved behavioral recovery by promoting the maturation of oligodendrocytes and enhancing remyelination. Mechanistically, when oligodendrocyte progenitors were cultured in differentiation medium, FBLN2 impeded their maturation into oligodendrocytes by engaging the Notch pathway, leading to cell death. Adeno-associated virus deletion of FBLN2 in astrocytes improved oligodendrocyte numbers and functional recovery in EAE and generated new myelin profiles after lysolecithin-induced demyelination. Collectively, our findings implicate FBLN2 as a hitherto unrecognized injury-elevated ECM, and a therapeutic target, that impairs oligodendrocyte maturation and myelin repair.

## Abstract 2

Targeting fibulin-2, an upregulated extracellular matrix component that inhibits myelin repair, improves recovery in models of multiple sclerosis.

## Graphical abstract

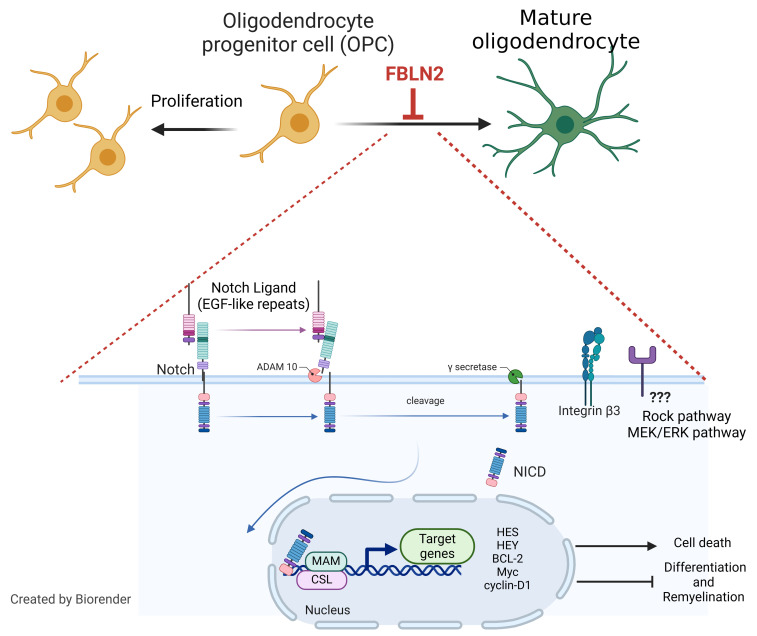

## Introduction

The importance of oligodendrocytes and myelin in the central nervous system (CNS) is evidenced by the neuronal dysfunction and physical disability observed following their damage (1). Oligodendrocyte loss and myelin impairment contribute not only to classic demyelinating disorders such as multiple sclerosis (MS) but also to dystonias, stroke, neurodegenerative conditions such as Alzheimer’s disease, and even cognitive dysfunctions (2–5). In preclinical studies, the generation of new oligodendrocytes and myelin is important for motor skill learning and memory (6, 7).

Remyelination is a repair response to myelin loss, and it protects denuded axons from irreversible degeneration as manifested by preserved axon density (8), decreased loss of thalamic volume (1), and low disability scores (9). Strategies to enhance remyelination such as overcoming impediments to repair are sought after (10, 11).

Remyelination begins with the recruitment and differentiation/maturation of oligodendrocyte progenitor cells (OPCs) to myelin-forming oligodendrocytes (10, 12). Remyelination may occur extensively at early stages of MS, but it often fails, contributing to progression of disability (9, 13, 14). While OPC numbers may not be reduced in many MS lesions, their maturation to oligodendrocytes and hence oligodendrocyte number are often deficient (11, 15). Identifying and overcoming factors that impair oligodendrocyte maturation are of interest.

One contributor to inefficient remyelination is excessive deposition of the extracellular matrix (ECM) in lesions (16). We described that versican-V1 deposited in MS lesions impedes OPC properties and remyelination (17). While there are more than 300 ECM core proteins, only a minority have been characterized in neurological injuries. Here, we began with investigations of proteome databases and then interrogated the roles and mechanisms of a hitherto unknown ECM component elevated in lesions of MS and its models, fibulin-2 (FBLN2).

## Results

### Prominent elevation of FBLN2 in CNS lesions.

To gain a thorough overview of the altered ECM in CNS injuries, we first interrogated available proteomic databases for MS and its animal model, experimental autoimmune encephalomyelitis (EAE). FBLN2 was qualitatively elevated in MS (18) and elevated 9.5-fold (19) in the quantitative EAE proteome library (Supplemental Figure 1A; supplemental material available online with this article; https://doi.org/10.1172/JCI176910DS1). As FBLN2 is minimally described in the CNS, with roles in synapse formation/neurogenesis (20) and elevated immunoreactivity but unknown functions in astrocytes after traumatic CNS injury (21), we sought to interrogate FBLN2 further.

**Figure 1:**
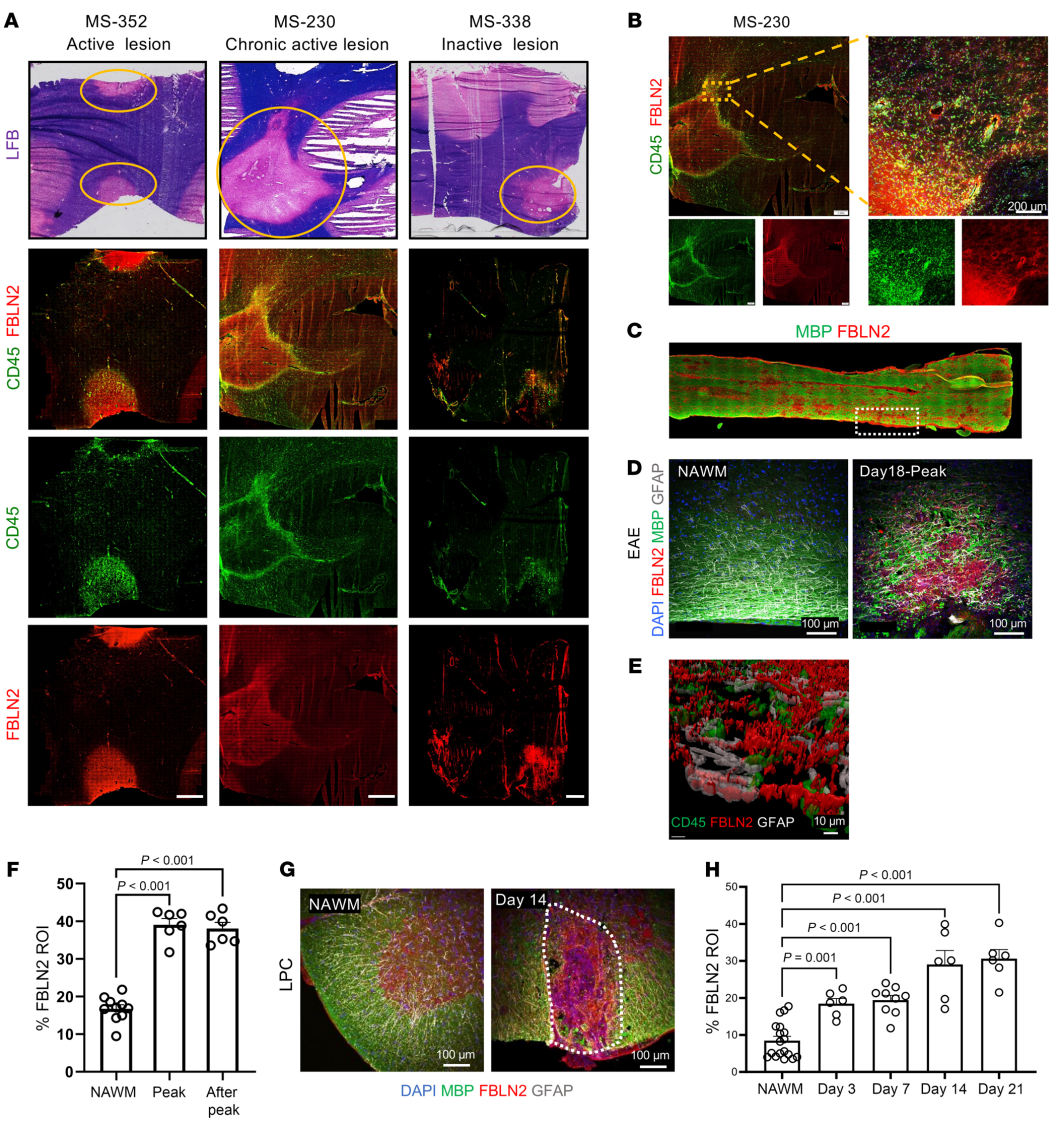
FBLN2 is upregulated in lesions of MS and its animal models. (**A**) Top: Luxol fast blue (LFB) and H&E histologically stained tissues showing demyelinated lesions from 3 MS brain samples. White matter lesions were defined by loss of LFB staining (yellow rings). Bottom: Immunofluorescence images labeled with CD45 for immune cells and FBLN2. Scale bars: 2 mm. (**B**) Yellow dotted square specifies the area shown at higher magnification to the right. Scale bars: 1 mm (left), 200 μm (right). (**C**) Representative image of large area of longitudinal section of spinal cord from EAE mice. White dotted box indicates a region of interest (ROI) tracked by loss of myelin. (**D**) Representative images of spinal cord sections from EAE mice comparing the normal-appearing white matter (NAWM) and lesion area. Scale bars: 100 μm. (**E**) Colocalization of FBLN2 in GFAP^+^ astrocytes but rarely in CD45^+^ immune cells using Imaris 3D rendering. Scale bar: 10 μm. (**F**) Quantification comparing the percentage of FBLN2 in NAWM and EAE lesions at different time points. *n* = 6 mice total per group. Data were acquired from 2 separate experiments, and each dot represents mean of 5 lesions analyzed per mouse. (**G**) Representative images of coronal sections of NAWM and LPC-induced lesion (dotted line) 14 days after injury. Scale bars: 100 μm. (**H**) Bar graph comparing the percentage of FBLN2 area within the LPC lesion over the time after injury. *n* = 6 mice for days 3, 14, and 21; *n* = 9 mice for day 7, from 2 separate experiments. Images in **A** and **C** were obtained by slide scanner. Images in **B**, **D**, **E**, and **G** were acquired by immunofluorescence laser confocal microscope (*Z*-stack). Data in **F** and **H** are presented as mean ± SEM; 1-way ANOVA, Bonferroni post hoc.

Frozen brain autopsy specimens from people with MS were analyzed for lesions by loss of luxol fast blue myelin staining and by prominent accumulation of CD45^+^ immune cells throughout the lesion (active) or in the hypercellular edge (chronic active lesion). FBLN2 immunoreactivity was markedly elevated in active and chronic active lesions, and in the inactive core, but not in normal-appearing white matter (Figure 1, A and B).

Next, we assessed FBLN2 expression in spinal cord white matter lesions in both EAE and lysophosphatidylcholine-induced (lysolecithin-induced) demyelination models. FBLN2 was accumulated in myelin basic protein–deficient (MBP-deficient) demyelinated EAE lesions at the peak (day 18) and post-peak (day 40) stages of clinical severity (Figure 1, C–F). In the CNS, FBLN2 is mainly expressed by endothelial cells and astrocytes (21, 22). Using Imaris 3D rendering, FBLN2 was found extracellularly or colocalized to glial fibrillary acidic protein–positive (GFAP^+^) astrocytes (Figure 1E). This was corroborated by in vitro data of increased FBLN2 in activated astrocytes (Supplemental Figure 1, B and C). Moreover, upregulation of FBLN2 persisted at different time points in lysophosphatidylcholine-demyelinated (LPC-demyelinated) lesions (Figure 1, G and H).

To evaluate whether FBLN2 is elevated across neurological disorders, we selected hemorrhagic stroke as a condition distinct from MS. In murine samples from collagenase-induced intracerebral hemorrhage and also in human hemorrhagic stroke, prominent accumulation of FBLN2 was noted (Supplemental Figure 1, E–G). Furthermore, interrogation of publicly available proteomic data sets revealed elevation of FBLN2 in Alzheimer’s disease (23), suggesting that FBLN2 upregulation is common in CNS injuries. While not highlighted by the original authors, our data mining shows higher levels of FBLN2 in the cerebrospinal fluid after severe traumatic brain injury and in people with Alzheimer’s disease compared with controls (24, 25).

### Course of EAE in FBLN2-deficient mice.

**Figure 2:**
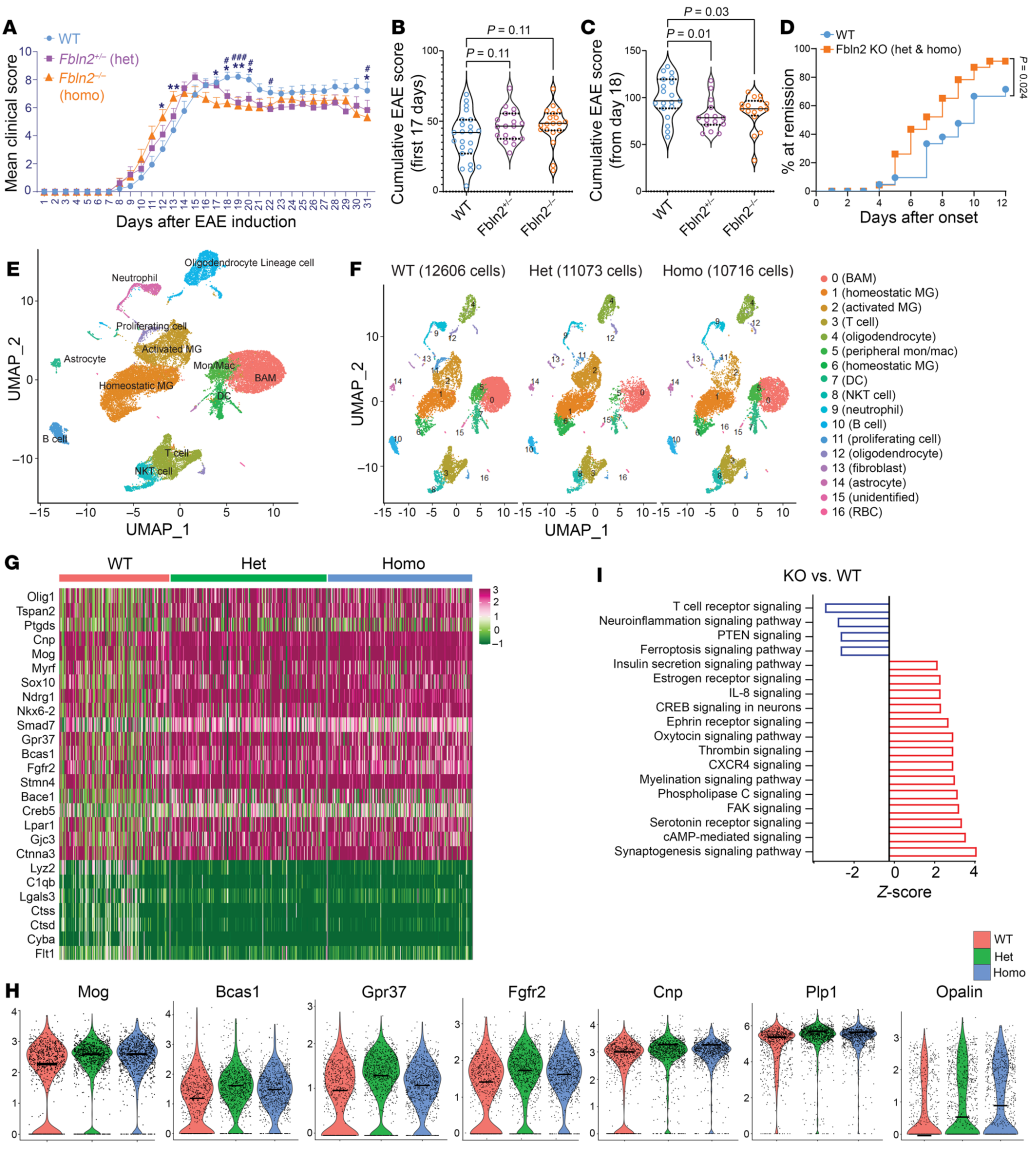
The better clinical recovery from EAE in FBLN2-deficient mice is associated with a profound myelination profile. (**A**) Average EAE clinical score (mean ± SEM) is shown. *n* = 23 mice for WT, 18 mice for *Fbln2*^+/–^, 19 mice for *Fbln2^–/–^*, pooled from 3 independent experiments; 2-way repeated-measures ANOVA (mixed-effects model), Dunnett’s multiple-comparison test; *P < 0.05, **P < 0.01, WT vs. *Fbln2^–/–^*; ^#^*P* < 0.05, ^###^*P* < 0.001, WT vs. *Fbln2^+/–^*. (**B** and **C**) Violin plots of cumulative EAE scores for the first 17 days (**B**) and from day 18 after induction (**C**) (median, solid black lines; interquartile range, dotted black lines). Kruskal-Wallis test with Dunn’s multiple comparisons. (**D**) Percentage of mice undergoing remission on each day from peak clinical severity. Curves were compared using the log-rank test. (**E** and **F**) Uniform manifold approximation and projection (UMAP) plot of 34,395 cells from spinal cords of 9 EAE mice (**E**) and across different experimental groups (**F**) depicting 17 clusters as determined by 30 principal components and 0.3 clustering resolution. (**G** and **H**) Heatmap (**G**) and violin plots (**H**) comparing the levels of select DEGs associated with remyelination in oligodendrocyte cluster. Midline in the plot is the median. (**I**) Select top activated (red) or inactivated (blue) pathways in oligodendrocytes from Het and Homo FBLN2-knockout spinal cords compared with WT spinal cords as predicted by IPA.

To better understand the role of FBLN2 in CNS injury and neuroinflammation, EAE was induced in 10- to 12-week-old C57BL/6 wild-type (WT) and FBLN2-deficient mice; the latter included heterozygous (*Fbln2^+/–^*) (Het) and homozygous (*Fbln2^–/–^*) (Homo) mice. The severity of EAE did not differ at the initial inflammatory stage of disease (Figure 2, A and B), likely because of the lack of activity of FBLN2 on immune responses, as explained later in this article. However, clinical disability scores improved significantly, albeit marginally, in FBLN2-deficient mice after the peak of clinical severity (Figure 2, A and C); this is the period in which regeneration of oligodendrocytes and myelin leads to a modest decline in EAE disability (as immune activity is still prominent) as noted by others (26). Notably, FBLN2-knockout mice recovered to remission faster than WT mice, where remission is defined as a reduction in clinical score from peak EAE severity by at least 0.5 points for 2 consecutive days or more (Figure 2D). These results highlight that FBLN2 deficiency facilitates recovery from EAE.

### Single-cell RNA sequencing links myelinating oligodendrocytes and FBLN2 inversely.

To delineate the underlying reasons for better functional recovery from EAE in FBLN2-deficient mice, single-cell RNA sequencing (scRNA-Seq) was performed on spinal cords from WT and FBLN2 Het and Homo knockout mice (3 mice per group) after peak EAE (day 18). Unsupervised integrated alignment of all 34,395 cells from the 9 spinal cords delineated 17 clusters that were present in all experimental groups (Figure 2, E and F). Cell population analyses revealed changes in cellular composition of spinal cords and, particularly, more oligodendrocytes in FBLN2-deficient mice compared with the WT group (Figure 2F). Lineage cell markers identified clusters 4 and 12 as oligodendrocyte-lineage cells (Supplemental Table 1 and Supplemental Figure 2, A and B). Oligodendrocytes from FBLN2-deficient mice displayed higher levels of myelination-related genes, including *Nrdg1*, *Myrf*, *Bcas1*, *Opalin*, *Gpr37*, *Smad7*, *Fgfr2*, *Cnp*, *Mog*, *Mbp*, *Mag*, and *Plp1* (Figure 2, G and H, and Supplemental Table 2).

Next, 523 differentially expressed genes (DEGs) in oligodendrocyte-lineage cells between FBLN2-deficient mice and WT were analyzed using Ingenuity Pathway Analysis (IPA; QIAGEN). Pathways such as ferroptosis and neuroinflammation were enriched in oligodendrocytes from WT mice, whereas myelination signaling and other pathways with pro-myelination properties, including cAMP-mediated, CREB, FAK/integrin, CXCR4, and IL-8 signaling, were enriched in FBLN2-deficient mice (27–30) (Figure 2I).

**Figure 3:**
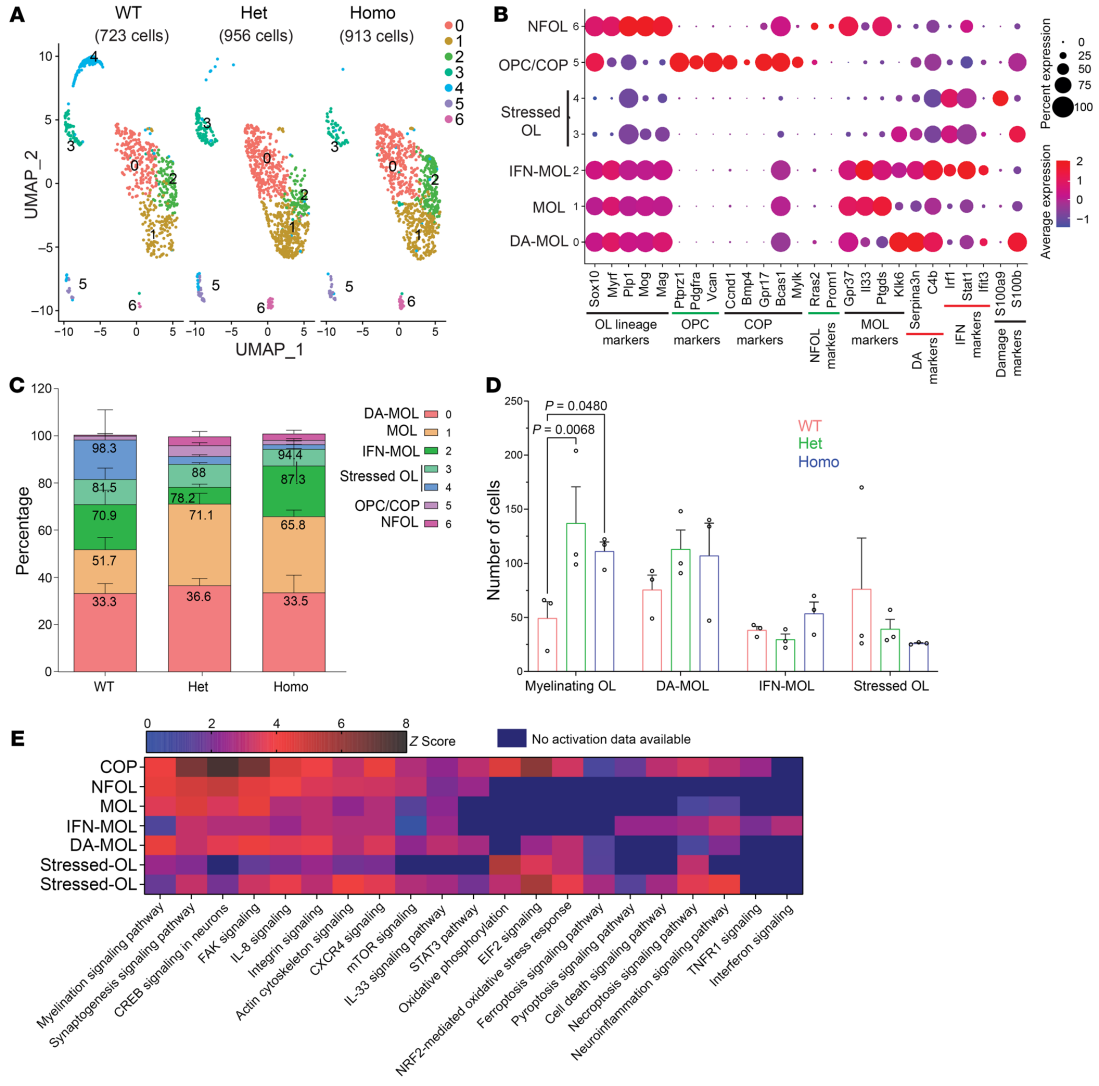
The frequency of distinct oligodendrocyte populations in EAE WT versus FBLN2-deficient mice. (**A**) UMAP plots of 2,592 oligodendrocyte-lineage cells reclustered into 7 distinct cell populations (principal components, 10; clustering resolution, 0.4). (**B**) Dot plot of representative marker genes enriched in oligodendrocytes. The size of the dot depicts percentage of cells expressing the gene in each cluster. The color represents the average gene expression level. (**C** and **D**) Bar graphs depicting percentage (**C**) and number (**D**) of cells in different subclusters of oligodendrocytes across groups (2-way repeated-measures ANOVA with Holm-Šídák post hoc test). (**E**) Heatmap showing the *z* scores of predicted pathways by IPA in oligodendrocyte subcluster. High and low *z* scores depict predicted activation and inhibition of pathways, respectively.

Oligodendrocyte-lineage clusters from the 3 experimental groups totaling 2,592 cells were subset and reclustered, demonstrating 7 subclusters (Figure 3A). Subclusters 0, 1, and 2 presented several markers of mature oligodendrocytes (MOLs) identified by previous studies (31, 32), including *Ptgds*, *Il33*, *Klk6*, *Myrf*, *Plp1*, *Mog*, and *Spock1*. Although these subclusters shared the same transcriptional profile, interferon (IFN) response genes (e.g., *Ifit3*, *Stat1*, and *Irf1*) and genes necessary for antigen presentation (*Psmb8/9*, *Tap1/2*) were enriched in subcluster 2 (IFN-MOLs). Unique expression of markers previously identified in disease-associated oligodendrocytes (DA-MOLs) was found in subcluster 0 (e.g., *C4b*, *Serpina3n*, *Plin3*, and *Anax5*). Subclusters 3 and 4 showed higher levels of inflammation and oxidative stress genes such as *S100b*, *S100a1/8/9*, *C1qa*, *Lyz2*, *Ftl*, *Tf*, and *Cyba/b* (Stressed-OLs). Subcluster 5 displayed OPC markers (*Sox6*, *Ptprz1*, *Pdgfra*, and *Vcan*) as well as markers of committed OPCs (COPs) such as *Bcas1*, *Gpr17*, and *Mylk*. Subcluster 6 was annotated as newly formed oligodendrocytes (NFOLs), as they expressed *Prom1*, *Rras2*, *Bcas1*, and higher levels of myelin genes (Figure 3B; Supplemental Figure 2, C and D; and Supplemental Table 3).

MOLs and DA-MOLs constituted the majority of the oligodendrocyte population in FBLN2-deficient mice, while Stressed-OLs were more abundant in WT mice (Figure 3C). The total number of COPs, NFOLs, and MOLs, which together represent myelinating oligodendrocytes, was significantly higher in FBLN2-knockout mice (Figure 3D).

COP, NFOL, and MOL subclusters (subclusters 5, 6, and 1) were predicted by IPA to be involved in myelination through upregulation of synthesis of lipid, organization of cytoskeleton, synaptogenesis, axon ensheathment, and other pathways such as CREB and mTOR signaling (Figure 3E and Supplemental Figure 2E). On the other hand, DA-MOLs, IFN-MOLs, and Stressed-OLs (subclusters 0, 2, 3, and 4) had an altered gene expression pattern in the modules predicted to promote cell death, ferroptosis, oxidative stress, and neuroinflammation (Figure 3E). Our findings highlight the importance of COPs, NFOLs, and MOLs as myelinating oligodendrocytes that were expanded in FBLN2-deficient mice with better clinical recovery from EAE. In conclusion, these findings uncovered a profound myelination profile in FBLN2-knockout mice during neuroinflammation.

### FBLN2-deficient mice have more mature oligodendrocytes in EAE and LPC injuries.

**Figure 4:**
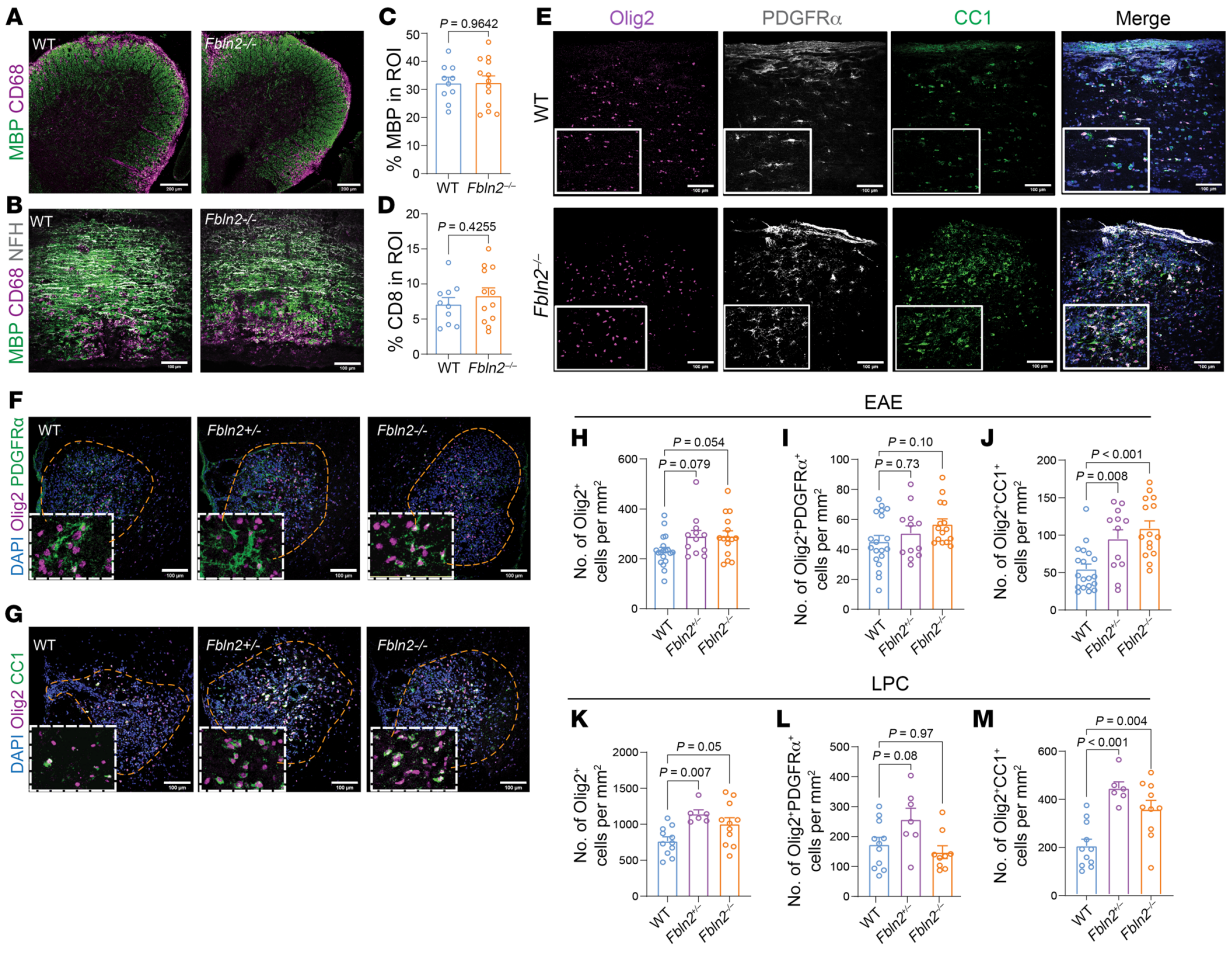
FBLN2 deficiency increases number of mature oligodendrocytes in demyelinating lesions. (**A** and **B**) Representative images of coronal (**A**) and longitudinal (**B**) sections of spinal cord from EAE mice comparing WT and *Fbln2^–/–^* mice. Tissues were stained for MBP and CD68. (**C** and **D**) Bar graphs comparing the percentage of ROI that is MBP^+^ (**C**) or CD68^+^ (**D**) (*n* = 10–12 mice; 2-tailed unpaired Student’s *t* test). (**E**–**G**) Representative images of longitudinal sections of spinal cord from EAE mice (**E**) and coronal sections of LPC lesions 14 days post injection (dpi) (**F** and **G**) stained for DAPI, Olig2, PDGFRα, and CC1. (**H**–**M**) Bar graphs comparing the number of Olig2^+^ lineage cells, Olig2^+^PDGFRα^+^ OPCs, and Olig2^+^CC1^+^ mature oligodendrocytes per square millimeter within EAE (**H**–**J**) and LPC (**K**–**M**) lesions. *n* = 19 mice for WT, 12 mice for *Fbln2*^+/–^, and 15 mice for *Fbln2^–/–^* over 3 separate EAE experiments; *n* = 11 mice for WT, 7 mice for *Fbln2*^+/–^, and 10 mice for *Fbln2^–/–^* over 2 separate LPC experiments; 1-way ANOVA, Bonferroni post hoc. All images were acquired by immunofluorescence laser confocal microscope (*Z*-stack). Scale bars: 200 μm (**A**) 100 μm (**B**, **E**, **F**, and **G**). Data are presented as mean ± SEM.

FBLN2 deficiency did not impact the extent of demyelination, inflammation, and immune cell infiltration at the peak phase of EAE (Figure 4, A–D, and Supplemental Figure 3, A–I), consistent with the comparable EAE severity during the initial inflammatory stage of the disease (Figure 2, A and B). This was further supported by the lack of obvious effects of FBLN2 on immune cells, as indicated by macrophage/microglia cytokine production or T cell proliferation in culture (Supplemental Figure 4, A–F).

Despite the similar degree of demyelination and inflammation, FBLN2 Homo and Het knockout mice showed an increase in number of mature oligodendrocytes (Olig2^+^CC1^+^) within lesions (EAE mice from Figure 2A at sacrifice). The number of OPCs (Olig2^+^PDGFRα^+^) remained unchanged (Figure 4, E and H–J).

Analyses were conducted in the LPC model as it shows localized and distinct phases of de- and remyelination at expected time points, facilitating the study of remyelination. In corroboration of the EAE results, coronal spinal cord sections analyzed 14 days after LPC demyelination showed that FBLN2-deficient mice had a higher number of mature oligodendrocytes within the lesion while OPC numbers remained the same (Figure 4, F, G, and K–M). Loss of FBLN2 did not alter the extent of inflammation and myelin content in LPC lesions, as identified by staining for immune cell markers or MBP (Supplemental Figure 3, J–L). MBP staining may not be the most accurate indicator of remyelination, as it stains both intact myelin and degraded myelin that has not yet been cleared by phagocytes. Collectively, findings from immunofluorescence staining of both EAE and LPC lesions confirm the scRNA-Seq data, indicating that the absence of FBLN2 leads to an increase in the number of mature oligodendrocytes following demyelination.

### FBLN2 kills differentiating OPCs.

**Figure 5:**
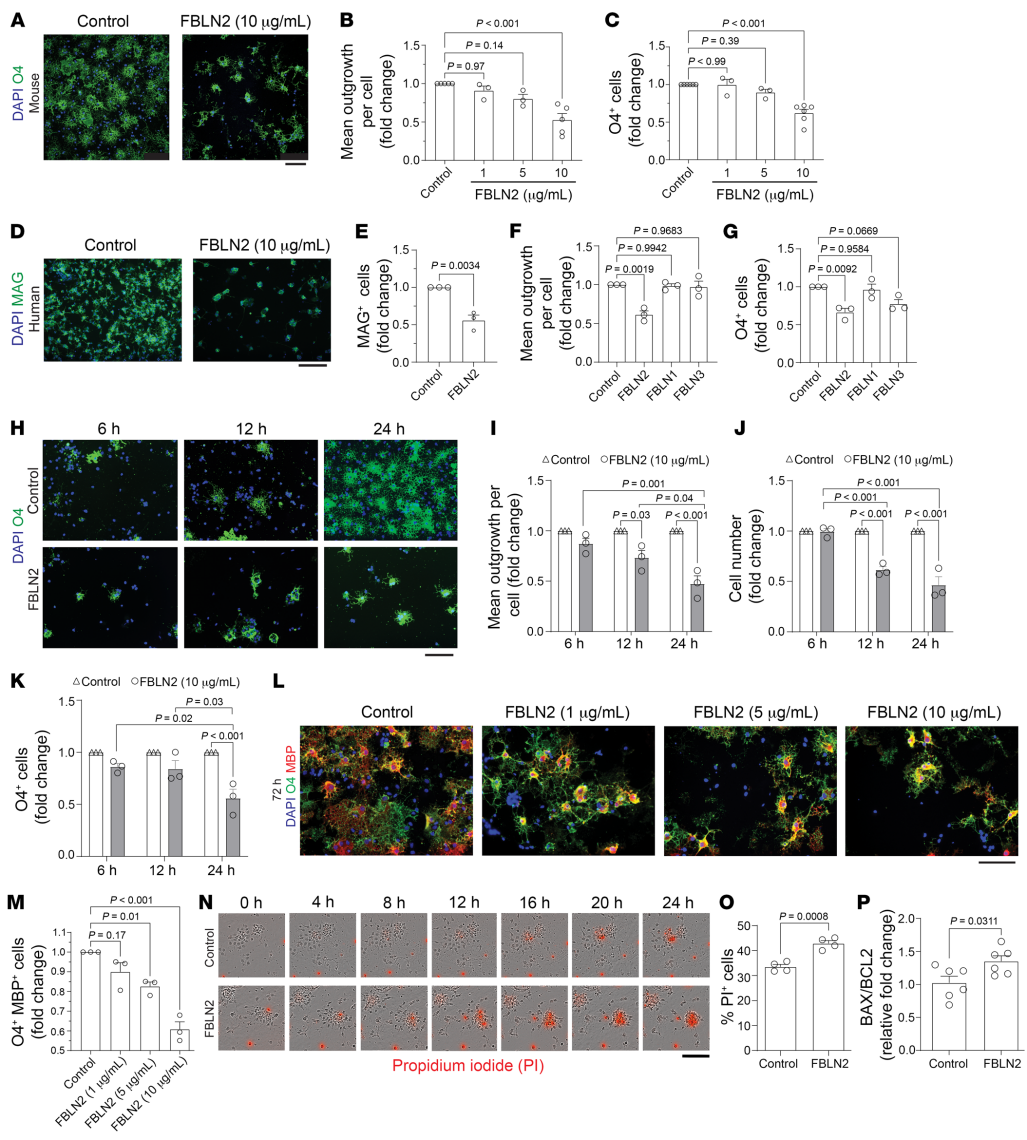
FBLN2 impairs maturation of oligodendrocytes and induces cell death. (**A**) Mouse OPCs stained for O4 24 hours after plating onto PBS (control) or FBLN2. (**B** and **C**) Fold change in mean process outgrowth (**B**) and number of O4^+^ cells (**C**) of mouse OPCs cultured on control and different concentrations of FBLN2 for 24 hours. *n* = 5 independent experiments for FBLN2 (10 μg/mL), *n* = 3 for FBLN2 (1 and 5 μg/mL); 1-way ANOVA, Bonferroni post hoc. (**D** and **E**) Representative images (**D**) and number of MAG^+^ cells (**E**) from human OPCs cultured on control and FBLN2 (10 μg/mL). *n* = 3 independent experiments; 2-tailed, unpaired Student’s *t* test. (**F** and **G**) Mean process outgrowth (**F**) and number of O4^+^ cells (**G**) of mouse OPCs cultured on coated wells with different members of FBLN family (10 μg/mL). *n* = 3 independent experiments; 1-way ANOVA, Bonferroni post hoc. (**H**–**K**) Mouse OPCs (**H**) and fold change in mean process outgrowth (**I**), number of total cells (**J**), and number of O4^+^ cells (**K**) at 6, 12, and 24 hours after plating onto control or FBLN2 (10 μg/mL). *n* = 3 independent experiments; 2-way ANOVA, Bonferroni post hoc. (**L**) Representative images of mature mouse oligodendrocytes stained for O4 and MBP at 72 hours. (**M**) Quantification comparing number of O4^+^MBP^+^ cells. *n* = 3 independent experiments; 1-way ANOVA, Bonferroni post hoc. (**N**) Live imaging of mouse OPCs plated on control and FBLN2 (10 μg/mL) in the presence of propidium iodide (PI) at different time points. (**O**) Proportion of PI^+^ OPCs after 24 hours. *n* = 3 independent experiments; 2-tailed, unpaired Student’s *t* test. Each experiment (dot) included 3–4 replicates. (**P**) Ratio of *Bax* to *Bcl2* mRNA expression in mouse OPCs using real-time PCR. *n* = 6 replicates over 2 separate experiments; 2-tailed, unpaired Student’s *t* test. Scale bars: 100 μm. Data are presented as mean ± SEM.

To define the properties of FBLN2 on oligodendrocytes, we isolated and plated OPCs onto a FBLN2 substrate in differentiation medium (without PDGF and FGF proliferation factors). While it did not affect initial adhesion, FBLN2 was strongly inhibitory of maturation of both human and mouse OPCs to oligodendrocytes. As OPCs differentiate, they extend their processes as a prerequisite for myelin enwrapping of axons in vivo, and they upregulate expression of sulfatide O4 and myelin proteins (e.g., MBP, MAG) while downregulating PDGFRα (10). FBLN2 reduced process outgrowth and proportion of O4- and MBP-expressing cells (Figure 5, A–E, L, and M). In contrast, other members of the FBLN family, including FBLN1 and FBLN3, were not inhibitory (Figure 5, F and G).

Live imaging of OPCs showed that the initial attachment and process extension of OPCs were not affected by plating onto FBLN2; however, subsequent cell density, maturation to O4^+^ oligodendrocytes, and process outgrowth declined noticeably after 12 hours (Figure 5, H–K, and Supplemental Figure 4, G and H). In contrast to oligodendrocytes, FBLN2 did not affect survival of microglia, astrocytes, and neurons (Supplemental Figure 4, A, B, and I–N).

To address whether FBLN2 hinders OPC proliferation, cells were grown in proliferation medium supplemented with growth factors necessary for OPC proliferation (PDGF and FGF). Dividing OPCs were labeled with 5-ethynyl-2′-deoxyuridine (EdU) for 6 hours before detection of the incorporated EdU. The percentage of proliferating OPCs did not change in the presence of FBLN2 (Supplemental Figure 5, A and B). Strikingly, when OPCs were cultured in differentiation medium so that they could mature to oligodendrocytes, FBLN2 induced a G_0_/G_1_-phase cell cycle arrest detected by flow cytometry (Supplemental Figure 5, C and D).

To examine whether FBLN2 induces apoptosis in differentiating OPCs possibly due to disrupted maturation program, live imaging of OPCs plated on control and FBLN2 substrates was performed in the presence of propidium iodide (PI) in the differentiation culture medium. Few OPCs incorporated PI as a sign of cellular compromise during the first hours of observation, whereas PI uptake was significantly increased in FBLN2-exposed cells after 12 hours (Figure 5, N and O). Moreover, real-time PCR gene expression analysis of pro-apoptotic *Bax* and anti-apoptotic *Bcl2* molecules from differentiating OPCs showed an increase in the *Bax*/*Bcl2* ratio following FBLN2 exposure for 6 hours (Figure 5P).

Collectively, these results highlight that FBLN2 does not affect OPC proliferation, but it induces cell death in differentiating (committed) OPCs.

### FBLN2 engages Notch signaling in oligodendrocytes, leading to a differentiation block followed by cell death.

**Figure 6:**
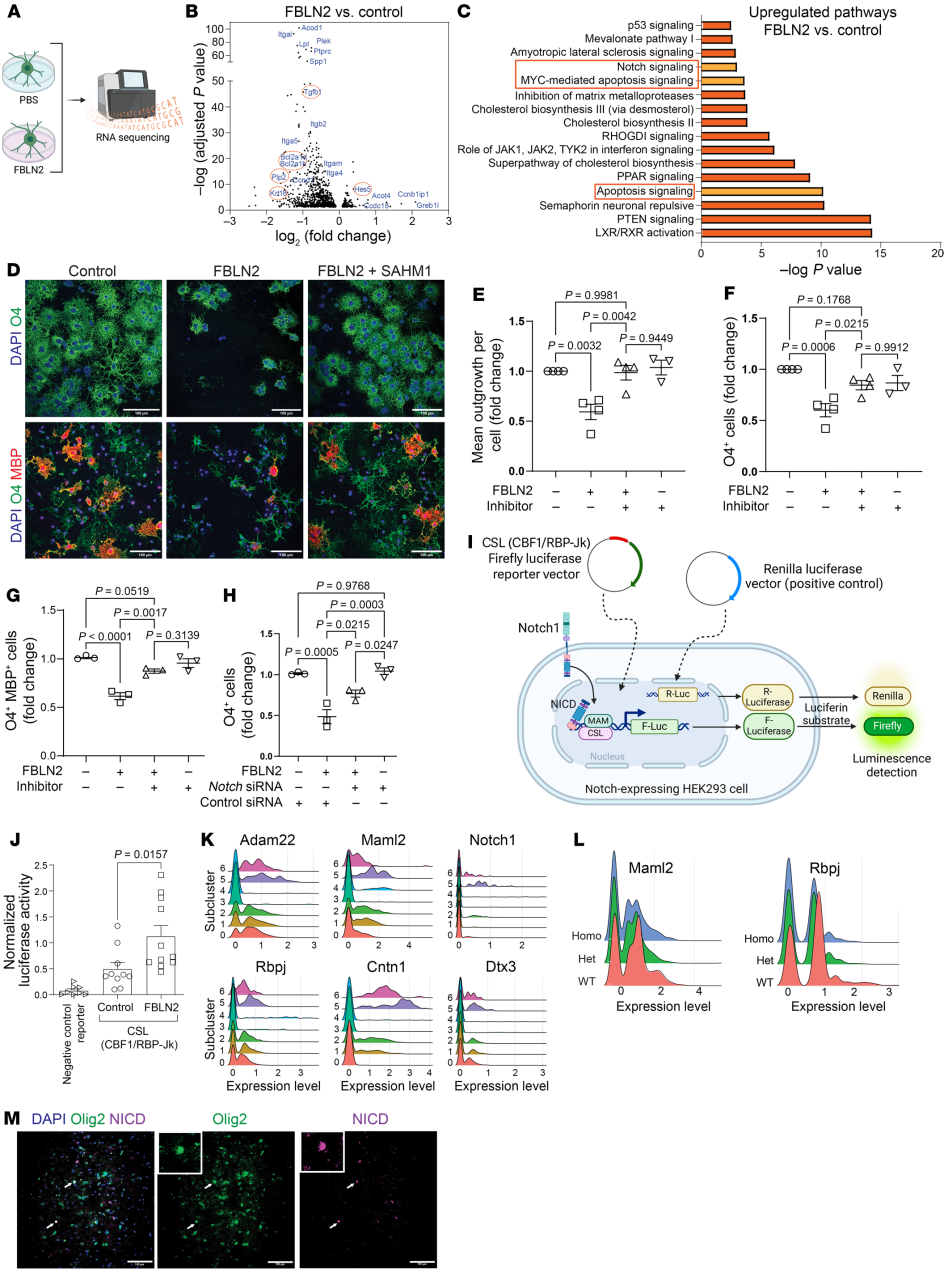
Blocking Notch pathway rescues the inhibitory effect of FBLN2 on oligodendrogenesis. (**A**) Schematic diagram of experimental design. Created with BioRender. (**B**) Volcano plots showing upregulated or downregulated genes in mouse OPCs cultured on FBLN2-coated (10 μg/mL) wells for 6 hours, identified by RNA sequencing. (**C**) Top upregulated pathways predicted by IPA from DEGs. RNA-sequencing data were acquired from 3 replicates per group (PBS or FBLN2). (**D**) Representative images of mouse OPCs cultured on control and FBLN2-coated (10 μg/mL) wells with or without Notch inhibitor (SAHM1, 10 μM) after 24 hours (top) and 72 hours (bottom). (**E** and **F**) Quantification for mean process outgrowth (**E**) and number of O4^+^ cells after 24 hours (**F**) (*n* = 4 independent experiments). (**G**) Number of O4^+^MBP^+^ cells at 72 hours (*n* = 3 independent experiments). (**H**) Number of O4^+^ cells 24 hours after transfection with 2 *Notch1* siRNAs (200 nM each; *n* = 3 independent experiments). Each experiment (dot) in **E**–**H** included 3–4 replicates; 1-way ANOVA, Tukey’s post hoc. (**I**) Schematic depicting the Notch pathway reporter assay using the constitutively expressing Renilla luciferase vector (positive control) and CSL (CBF1/RBP-Jκ) firefly luciferase reporter vector. (**J**) Bar graph comparing relative luciferase activity (firefly to Renilla) in HEK239 cells transfected with luciferase reporter or negative control vectors in the presence or absence of 10 μg/mL FBLN2 (*n* = 8–10 replicates over 2 separate experiments; 1-way ANOVA, Bonferroni post hoc). (**K** and **L**) Ridge plots comparing expression of select genes involved in Notch pathway across oligodendrocyte subclusters (0, DA-MOL; 1, MOL; 2, IFN-MOL; 3 and 4, Stressed-OL; 5, COP; 6, NFOL) (**K**) and experimental groups (**L**). (**M**) Immunofluorescence images of MS brain sample labeled for Olig2 and NICD. White arrows show the presence of Notch signaling in oligodendrocytes. Scale bars: 100 μm, insets, original magnification, 2×. Data are presented as mean ± SEM.

To investigate the mechanisms of FBLN2, mouse OPCs were plated in control or FBLN2-coated wells in differentiation medium for 6 hours. Bulk RNA sequencing was performed to identify up- or downregulated genes (Figure 6, A and B). IPA analysis on DEGs by FBLN2-exposed OPCs highlighted the enrichment of apoptosis and Notch signaling pathways, while cell cycle regulation and actin/microtubule cytoskeleton signaling were downregulated (Figure 6C and Supplemental Figure 5E). FBLN2 exposure inhibited various myelination-related signaling pathways known to promote oligodendrogenesis, including mTOR, TGF-β, PI3K/AKT, MAPK, IL-8, and CXCR4 pathways (27–30, 33) (Supplemental Figure 5E). Several cell cycle–related genes as well as pro- or antiapoptotic genes such as *Ccnd1*, *Caspase-9*, *Krt18*, *Birc3*, *Timp-1*, and *Cflar* were dysregulated in FBLN2-exposed OPCs (Supplemental Figure 5F); these contributed to the death observed above when OPCs were differentiating to oligodendrocytes in the presence of FBLN2.

The RNA-Seq data set showed that Notch signaling was enriched in differentiating OPCs cultured on FBLN2 substrate (Figure 6C). Notch proteins are transmembrane proteins activated by ligands such as Jagged1. Ligand engagement results in the proteolytic cleavage of the Notch receptor and release of its intracellular domain NICD that translocates to the nucleus to induce expression of target genes such as *Hes5* (34). Notch signaling regulates cell proliferation, differentiation, and apoptosis in a cell context–dependent manner. While it promotes the differentiation of most glial cell subtypes, Notch pathway inhibits oligodendrocyte maturation (34, 35). Thus, we hypothesized that FBLN2 activates Notch signaling in differentiating OPCs, resulting in maturation block and subsequently cell death. In support, pharmacological inhibition of Notch signaling (SAHM1, 10 mM, 24 hours) overcame FBLN2-mediated reduction in process outgrowth and maturation of oligodendrocytes, as evidenced by O4 and MBP expression (Figure 6, D–G). In addition, genetic manipulation using 2 *Notch1* siRNAs (200 nM) reversed the differentiation block of FBLN2 and increased the number of O4^+^ oligodendrocytes after 24 hours (Figure 6H). In accordance with RNA-Seq findings, higher levels of NICD were shown in OPCs plated on FBLN2 substrate using Western blot analysis (Supplemental Figure 5, G and H). Finally, we used a Notch pathway luciferase reporter assay to measure functional Notch activity in response to FBLN2. Relative luciferase activity (firefly to Renilla luminescence) increased significantly in HEK239 cells transfected with CSL (*CBF1*/*RBP-Jk*) luciferase reporter following FBLN2 (10 μg/mL) treatment, validating Notch activation by FBLN2 (Figure 6, I and J).

The tissue culture results were corroborated in vivo. According to the scRNA-Seq data set, Notch signaling was present in the COP subcluster that can be considered equivalent to differentiating OPCs in culture (Figure 6K). Some Notch-related genes (*Maml2*, *Rbpj*) showed an upward trend in WT mice (Figure 6L); however, it did not reach statistical significance owing to the small size of this population especially in WT mice. Notch activation in oligodendrocytes of MS lesions was confirmed by immunolabeling for NICD (Figure 6M). Immunofluorescence staining of spinal cord from EAE mice as well as LPC lesions showed a smaller population of oligodendrocytes expressing HES5 or cleaved caspase-9 in FBLN2-deficient mice (Supplemental Figure 6, A–I). Moreover, less Notch activity and lower amount of cleaved caspase-9 were detected by Western blot analysis of spinal cord lysates from FBLN2-knockout mice (Supplemental Figure 6, J–M).

While our findings suggest that FBLN2 impairs oligodendrogenesis through engaging the Notch signaling pathway, the involvement of other pathways is not ruled out. When OPCs were exposed to inhibitors of several signaling pathways known to affect oligodendrogenesis (27, 33), we found that an inhibitor of ROCK and MEK/ERK, but not of p38 MAPK, PI3K/AKT/mTOR, Wnt/β-catenin, Src/Syk, BMP4, or Smad3 signaling, rescued the FBLN2 inhibition of process formation at 24 hours (Supplemental Figure 7, A–H).

### Reduction of FBLN2 levels by astrocyte-targeted AAV promotes oligodendrogenesis and myelin profiles.

**Figure 7:**
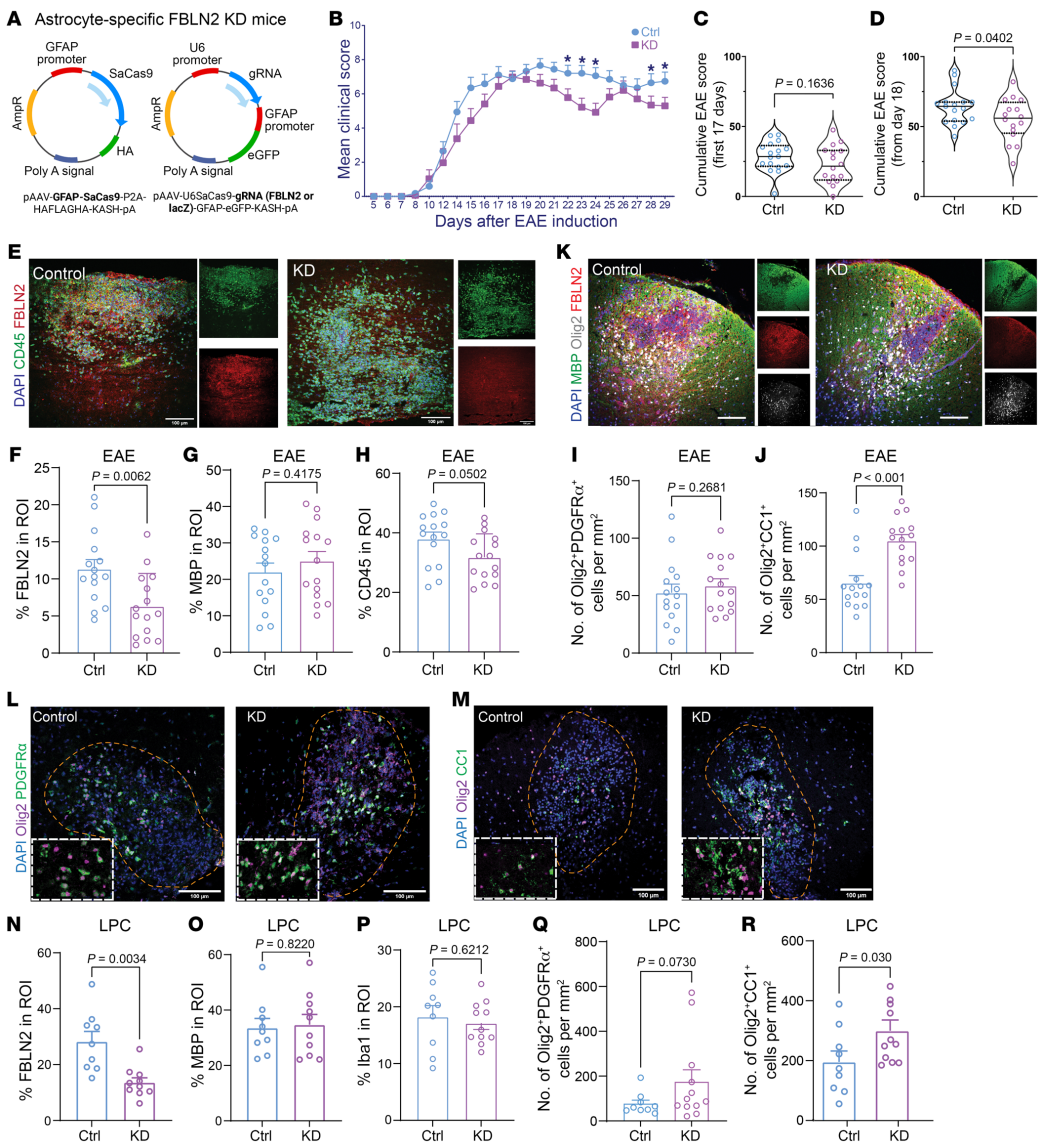
Astrocytic deletion of FBLN2 improves functional recovery in EAE, which is associated with more robust oligodendrogenesis. (**A**) Schematic showing the genome of AAVs used in this study. AAVs were injected 2 weeks before EAE induction or LPC surgery for targeted disruption of FBLN2 (KD) or nontarget guide RNA. (**B**) Average EAE clinical score. (**C** and **D**) Violin plots comparing the cumulative EAE scores for the first 17 days (**C**) and from day 18 (**D**). Solid black lines represent medians; quartiles are shown by dotted black lines. *n* = 17 mice for Ctrl, 15 mice for KD from 3 independent experiments; Mann-Whitney test; *P < 0.05. (**E**–**H**) Representative images of longitudinal spinal cord sections from EAE mice (**E**) and quantifications for FBLN2 (**F**), MBP (**G**), and CD45 (**H**) percentage area of the lesion. (**I** and **J**) Graphs comparing number of Olig2^+^PDGFRα^+^ OPCs (**I**) and Olig2^+^CC1^+^ mature oligodendrocytes (**J**) per square millimeter of EAE lesions. *n* = 15 mice per group over 3 separate experiments; 2-tailed unpaired Student’s *t* test. (**K**–**R**) Representative images of LPC lesions 14 dpi (**K**–**M**) and quantifications comparing percentage area of FBLN2 (**N**), MBP (**O**), Iba1 (**P**), and number of OPCs (**Q**) or mature oligodendrocytes (**R**) per square millimeter of lesion ROI. *n* = 9 mice for Ctrl, 11 mice for KD over 3 separate experiments; 2-tailed unpaired Student’s *t* test. Images were acquired by immunofluorescence laser confocal microscope (*Z*-stack). Scale bars: 100 μm. The insets are magnified ×1.7. Data are presented as mean ± SEM.

To overcome the effects of injury-induced elevation of FBLN2 in the CNS, an adeno-associated virus–coupled (AAV-coupled) CRISPR/Cas9 system was used to target its expression in astrocytes given the expression of FBLN2 in this population (Figure 1E and Supplemental Figure 1, B and C). We generated 2 different AAVs: (a) recombinant PHP.eB AAV vector packaging a specific guide RNA for targeted disruption of FBLN2 (U6-Fbln gRNA-GFAP-eGFP), and (b) recombinant PHP.eB AAV vector encoding Cas9 under the control of a GFAP promoter (GFAP-SaCas9-HAFLAGHA) (Figure 7A). AAVs (3 × 10^11^ viral genomes [vg] per virus) were codelivered retro-orbitally to mice 2 weeks before EAE induction or LPC injection. Mice were injected with AAV encoding nontarget guide RNA (U6-Luc gRNA-GFAP-eGFP) as control. Transduction efficiency ranged between 30% and 40% in both astrocyte culture and in vivo experiment (Supplemental Figure 8, A–D). Our data showed the specificity, feasibility, and success of this approach to lower FBLN2 levels in vitro and in vivo (Supplemental Figure 8, E–J, and Figure 7, F and N). Astrocyte-specific FBLN2 knockdown reduced average EAE daily score after the peak of clinical severity (Figure 7, B–D). Although the extent of demyelination and inflammation remained unchanged, astrocytic deletion of FBLN2 increased the number of mature oligodendrocytes in EAE or LPC lesions (Figure 7, E–R).

**Figure 8:**
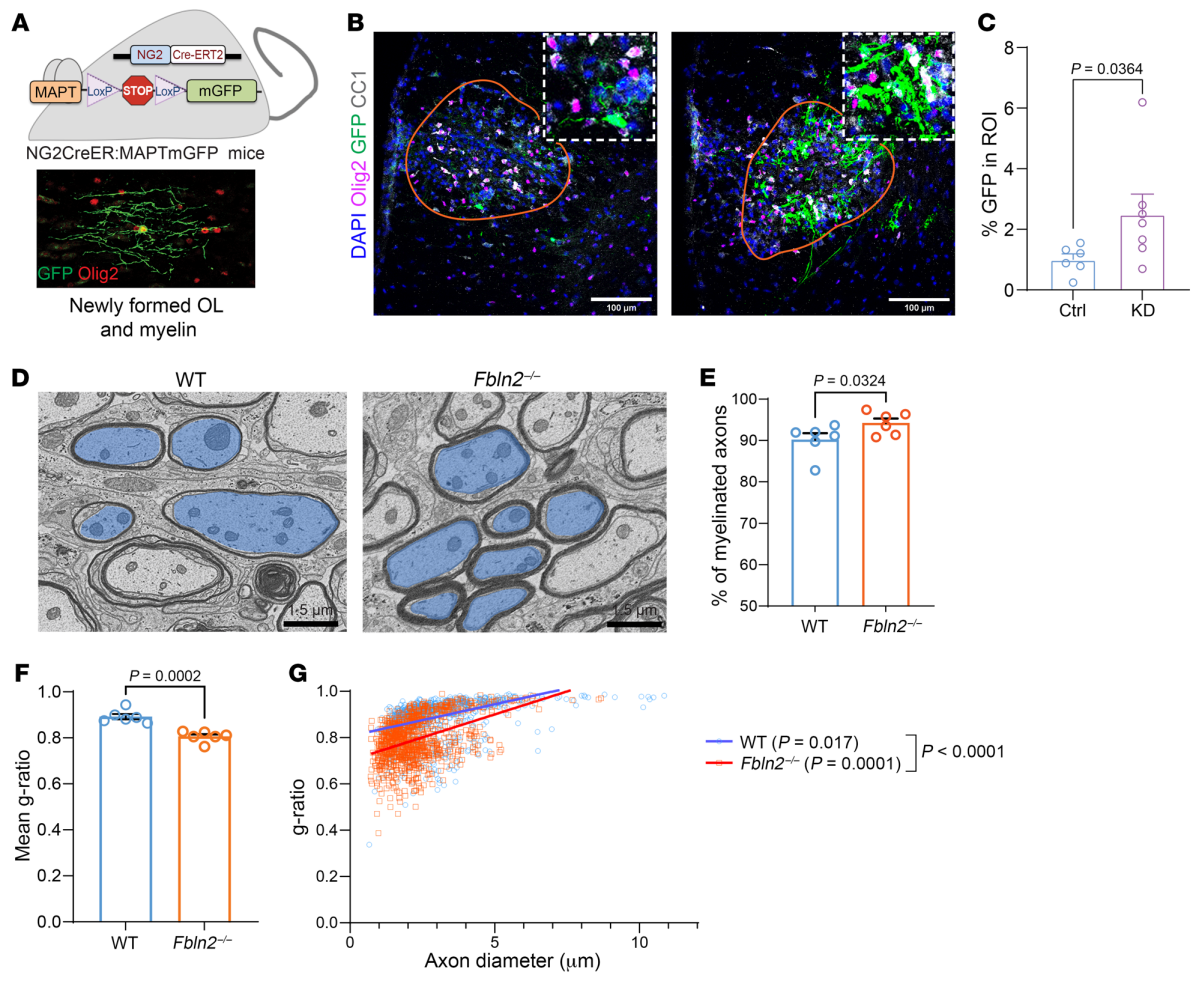
FBLN2 deficiency enhances remyelination. (**A**) NG2^CreER^ MAPT^mGFP^ mice were used to identify newly formed oligodendrocytes and myelin as GFP^+^. (**B**) Representative images of LPC lesions 14 dpi from NG2^CreER^ MAPT^mGFP^ mice that had received AAVs 2 weeks before surgery. Images were acquired by immunofluorescence laser confocal microscope (*Z*-stack). Scale bars: 100 μm. The insets are magnified ×1.7. (**C**) Bar graph showing the extent of GFP in lesions (*n* = 6 mice over 2 separate experiments; 2-tailed, unpaired Student’s *t* test). (**D**) Electron micrographs of LPC-induced lesions from WT and *Fbln2*^–/–^ mice at 14 dpi. Blue pseudocolored regions indicate examples of axons from myelinated axons. Scale bars: 1.5 μm. (**E**) Dot plot of percentage remyelinated axons. (**F** and **G**) Mean g-ratio (**F**) and scatterplot of g-ratio (*y* axis) in relation to axon diameter (*x* axis) (**G**) of individual fiber (*n* = 150 axons per mouse, 6 mice per group; simple linear regression of slopes). **E**–**G**: *n* = 6 mice per group; 2-tailed, unpaired Student’s *t* test; mean ± SEM.

Finally, to identify newly formed oligodendrocytes and myelin sheaths, NG2^CreER^ MAPT^mGFP^ mice were used, in which GFP expression indicates new oligodendrocytes and myelin (7, 17, 26) (Figure 8A). NG2^CreER^ MAPT^mGFP^ mice received AAVs 2 weeks before LPC injection. GFP cassette was removed from the AAV vector to avoid interference with GFP signal from newly formed oligodendrocytes. FBLN2 deletion in astrocytes resulted in larger area of GFP within the LPC lesions 14 days after injection, and cells with profuse myelin profiles could be observed (Figure 8, B and C). Thus, reducing lesional FBLN2 improves oligodendrogenesis, which is associated with better functional recovery in EAE and new myelin profiles in toxin-induced injury.

### Loss of FBLN2 improves remyelination.

We used blinded electron microscopy analyses to corroborate that FBLN2 deletion promotes remyelination after 14 days of LPC-induced demyelination. As remyelination frequently occurs at the lesion border, our region of interest included the inner border of the lesion, between normal-appearing white matter and lesion core (Supplemental Figure 8K). This region was characterized by a greater proportion of myelinated axons compared with the completely demyelinated lesion center, as well as by thinner myelin sheaths typical of remyelination compared with normal-appearing white matter outside of the lesion (Supplemental Figure 8L).

First, we addressed axonal density as an indicator of preservation of axons after demyelination. While total axon density showed a trend toward a higher level in FBLN2-deficient mice, these differences did not reach statistical significance. However, FBLN2-knockout mice had a higher proportion of remyelinated axons following injury (Figure 8, D and E). Next, we used g-ratio analysis (diameter of axon divided by diameter of axon plus myelin) assessing the thickness of myelin sheath in relation to the diameter of axon, where a ratio of 1 is fully demyelinated. Compared with WT, FBLN2-knockout mice showed a decrease in average g-ratio from 0.89 to 0.80, with significantly different linear regression lines (Figure 8, F and G). These findings indicate an increase in myelin thickness in remyelinated axons in FBLN2-knockout mice. Overall, these results emphasize that loss of FBLN2 facilitates remyelination.

## Discussion

The altered ECM microenvironment of CNS lesions is a relatively understudied area, even though ECM components affect cells in profound ways. Here, we implicate an understudied ECM component existent in CNS lesions with prominent inhibitory effects on maturation of oligodendrocytes from OPCs, an important step in remyelination to restore functions in the CNS. However, we noticed the minor improvement in EAE disability scores in FBLN2-knockout mice. This can be attributed to the absence of direct effects of FBLN2 on immune cells. Moreover, in contrast to regenerative compounds such as clemastine, lack of FBLN2 attenuates the inhibitory microenvironment lesion rather than directly targeting OPCs.

In our study, we did not find differences between *Fbln2^+/–^* and *Fbln2^–/–^* mice. Thus, the loss of a single allele overcame the defect in formation of mature oligodendrocytes after demyelination, which was not enhanced by loss of the second allele. This suggests that there are additional inhibitory molecules within the lesion, such as chondroitin sulfate proteoglycans, as well as compensatory mechanisms in response to complete loss of FBLN2, which masks the difference between heterozygous and homozygous mice. These remain to be addressed in future studies.

That FBLN2 engages Notch signaling in OPCs is significant, as Notch1 engagement by its ligand Jagged1 on axons of retinal ganglion cells prevents premature OPC maturation and thus controls the timely appearance of oligodendrocytes in optic nerve development (35). Notch1 signaling has been reported in MS lesions where Jagged1 of reactive astrocytes interacts with Notch1 on OPCs; this limited OPC differentiation to oligodendrocytes via the transcription factor HES5, which competes with SOX10 to block MBP expression (36). Subsequent studies showed that blocking Notch signaling attenuated EAE severity and improved myelin repair (37). Moreover, Notch deficiency in OPCs promoted remyelination in LPC and cuprizone models of demyelination (38, 39). To these results, we now add FBLN2 as an injury-enhanced trigger for Notch inhibition of OPC differentiation in MS and likely other neurological conditions. In summary, we have identified a hitherto unknown inhibitor of oligodendrogenesis and myelin repair. Overcoming FBLN2 has therapeutic potential not only in MS but also in other conditions in which myelin impairment contributes to axonal dysfunction and cognitive decline.

## Methods

### Sex as a biological variable.

Our study examined female mice since women are more susceptible to MS than men by a ratio of approximately 3:1. Besides, for the most uniform EAE development, use of female mice is recommended.

### MS specimens.

Postmortem frozen brain tissues from people with MS were obtained from the Multiple Sclerosis and Parkinson’s Tissue Bank situated at Imperial College, London, United Kingdom. Demyelinating lesions from the brains of 2 women (aged 50 and 42 years, MS-230 and MS-338) and 1 man (aged 43, MS-352) were analyzed for this study. The intracerebral hemorrhage (ICH) tissue came from a 70-year-old male with stroke. It was collected 3 days after ICH at the University of Calgary’s Foothills Hospital.

### Mice.

Fibulin-2–deficient (JAX 019895), NG2^CreER^ (JAX 008538), and MAPT^mGFP^ (JAX 021162) mice were acquired from The Jackson Laboratory and bred in the University of Calgary animal facility.

### Plasmid construction.

pJEP317-pAAV-U6SaCas9gNRA(SapI)-EFS-GFP-KASH-pA, pJEP312-pAAV-CMV-SaCas9-P2A-HAFLAGHA-KASH-pA (gifts from Jonathan Ploski, Addgene plasmids 113694 and 113689), and pAAV-GFP-eGFP (gift from Bryan Roth, Addgene plasmid 50473) were used. The EFS promoter of pJEP317-pAAV-U6SaCas9gNRA(SapI)-EFS-GFP-KASH-pA was excised by AgeI and Xba restriction enzyme digestion and replaced with the GFAP promoter sequence PCR-amplified from pAAV-GFAP-GFP to generate pAAV-U6-GFAP-GFP-KASH-pA using the NEBuilder hifi DNA assembly cloning kit (New England Biolabs). Likewise, the CMV promoter of pJEP312-pAAV-CMV-SaCas9-P2A-HAFLAGHA-KASH-pA was excised by digestion with XbaI and AgeI and replaced with the GFAP promoter sequence PCR. Potential single-guide RNAs (sgRNAs) with the SaCas9 PAM sequence (NNGRR) targeting the mouse fibulin-2 gene were designed using the Broad Institute GPP sgRNA designer. Complementary oligonucleotides with appropriate overhang sequences and 5′ phosphorylation modifications 5′-P-ACCGACCCTCCTGCATGACACTTCG-3′ and 5′-P-AACCGAAGTGTCATGCAGGAGGGTC-3′ were annealed and subcloned into BspQI-digested pAAV-U6-GFAP-GFP-KASH-pA. For the nontarget control, sgRNA targeting a lacZ sequence was used (40). All plasmid constructs were verified by restriction enzyme mapping and Sanger DNA sequencing.

### AAV production.

AAV viral vectors containing the PHP.eB capsid were generated using the methods of Challis et al. (41). PHP.eB capsid has been engineered to efficiently transduce the central nervous system (42). Briefly, 293FT cells (Thermo Fisher Scientific) were grown to about 90% confluence in hyperflasks (Corning) and cotransfected with 129 μg pHELPER (Agilent), 238 μg rep-cap plasmid encoding RH10 (gift from James M. Wilson, Addgene plasmid 112866), and 64.6 μg of transfer plasmid using the PEIpro transfection reagent (Polyplus). AAVs were precipitated from medium harvested after 3 days and 5 days using 40% PEG/2.5 M NaCl in buffer containing 500 mM NaCl, 40 mM Tris base, and 10 mM MgCl_2_. The lysate was incubated with 100 U/mL salt-active nuclease (Arcticzymes) at 37°C for 1 hour and then centrifuged at 2,000*g* for 15 minutes. AAV was purified from the resulting lysate using an iodixanol step gradient containing 15%, 25%, 40%, and 60% iodixanol in OptiSeal tubes (Beckman Coulter) followed by centrifugation at 350,000*g* using a Type 70 Ti ultracentrifuge rotor (Beckman Coulter). After centrifugation, the AAVs were harvested from the 40% layer using a 10 mL syringe and 16-gauge needle, diluted in 1× PBS containing 0.001% Pluronic F68 (Gibco), and filtered using a 0.2 μm syringe filter. The AAVs were concentrated and buffer-exchanged by 5 rounds of centrifugation using Amicon Ultra-15 100-kDa molecular weight cutoff centrifugal filter units (MilliporeSigma). The titer was determined using the qPCR Adeno-Associated Virus Titration kit (Applied Biological Materials), and the purity was verified by SDS-PAGE and total protein staining using InstantBlue reagent (Expedeon).

### EAE induction.

Littermate female WT, heterozygous (*Fbln2^+/–^*), and homozygous (*Fbln2^–/–^*) mice (10–12 weeks old) were injected subcutaneously with 50 μg MOG_35–55_ peptide (synthesized by Protein and Nucleic acid Facility, Stanford University, Stanford, California, USA) emulsified in CFA (Thermo Fisher Scientific) supplemented with 10 mg/mL heat-inactivated *Mycobacterium tuberculosis* H37Ra (MilliporeSigma). A total of 100 μL emulsion was injected at 2 sites into hind flanks. Pertussis toxin (300 ng per 200 μL; List Biological Laboratories, 180) was intraperitoneally injected on day 0 and 48 hours after MOG immunization. Clinical signs of EAE were monitored daily on a scale of 0–15 (17). Remission was defined as a reduction in clinical score from peak EAE disability by at least 0.5 points for 2 consecutive days or more. Mice were euthanized with ketamine (100 mg/kg) and xylazine (10 mg/kg) injected intraperitoneally and then perfused with PBS through the left ventricle of the heart.

### LPC-induced demyelination.

LPC was injected into the ventral spinal cord to induce experimental demyelination as described previously (43). To induce focal demyelination in the ventrolateral white matter of the spinal cord, 1% lysolecithin (MilliporeSigma, L1381) resuspended in 0.5 μL sterile PBS was injected at a rate of 0.25 μL/min over 2 minutes. A 10 μL Hamilton 34-gauge needle was inserted 1.3 mm into the ventral spinal cord between the T3 and T4 vertebrae. Mice were then sutured and monitored until recovery. The lower cervical and upper thoracic section of the spinal cord was dissected at 7, 14, and 21 days after injection.

### Collagenase-induced ICH.

The procedure for collagenase-induced ICH in mice was described in a previous study (44). Briefly, a 0.5 mm cranial burr hole was made with a microdrill in the skull above the right striatum according to the following coordinates: 2.0 mm lateral and 0.8 mm anterior to the bregma. Collagenase type VII (0.05 U) dissolved in 0.5 μL of saline was injected through the hole until 3.2 mm beneath the skull, ending in the right striatum. The injection (5 minutes) was controlled by a UMP3 UltraMicroPump (SMARTouch). The needle was kept inside the brain for another 5 minutes to prevent reflux before removal. Finally, the surgical site was sutured and disinfected.

### Immunofluorescence staining.

Spinal cords were postfixed overnight in 4% paraformaldehyde (PFA) at 4°C, then dehydrated in 30% sucrose solution for 72 hours. Tissues were then frozen in FSC 22 Frozen Section Media (Leica) and cut coronally or longitudinally into 20 μm sections using a cryostat (Thermo Fisher Scientific). Tissues were collected onto Superfrost Plus microscope slides (VWR) and stored at –20°C before staining.

Slides were thawed at room temperature for 15 minutes, then fixed with 4% PFA for 15 minutes. Blocking of samples was performed using horse serum blocking solution (0.01 M PBS, 10% horse serum, 1% BSA, 0.1% cold fish skin gelatin, 0.1% Triton X-100, and 0.05% Tween-20) for 1 hour at room temperature. Tissues were then incubated overnight at 4°C with diluted primary antibodies in antibody dilution buffer (PBS, 1% BSA, 0.1% cold fish skin gelatin, and 0.1% Triton X-100). After washing (3 times, 5 minutes) with PBS containing 0.1% Tween-20, slides were incubated with the fluorophore-conjugated secondary antibodies (1:400; Jackson ImmunoResearch Laboratories) and DAPI (1 μg/mL; MilliporeSigma) suspended in the antibody dilution buffer for 1 hour at room temperature. After washing (3 times, 5 minutes each), slides were mounted using Fluoromount G (Southern Biotech). Isotype controls and secondary antibody controls were included (Supplemental Figure 1D).

The following primary antibodies were used for immunofluorescence microscopy to identify specific targets: rabbit anti–mouse myelin basic protein (MBP) (1:200; Abcam, ab7349), rabbit anti–mouse/human Olig2 (1:200; MilliporeSigma, ab9610), goat anti–mouse PDGFRα (1:200; R&D, AF1062), mouse anti–mouse adenomatous polyposis coli (APC) (1:200; MilliporeSigma, clone CC-1, OP80), chicken anti-GFP (1:500; Aveslab, GFP-1020), rabbit anti–mouse Iba1 (1:500; Wako, 019-19741), rat anti–mouse CD45 (1:50; BD Pharmingen, 550539), rat anti–human CD45 (1:500; Invitrogen, MA5-17687), rat anti–mouse CD4 (1:100; BD Pharmingen, clone RM4-5, 56-0042-82), rabbit anti–mouse NF-H (1:1,000; Encor, RPCA-NF-H), goat anti–mouse/human GFAP (1:1,000; Novus, NB100-53809), rat anti–HA tag (1:500; Novus, NBP2-50416), rat anti–mouse CD68 (1:500; BioLegend, 137002), rabbit anti–mouse cleaved caspase-9 (1:200; Invitrogen, PA5-105271), rabbit anti–mouse Hes5 (1:200; Abcam, ab25374), and rabbit anti–mouse/human fibulin-2 (1:100; Invitrogen, catalog PA5-75510 and catalog PA5-79239). Images were captured on the Leica TCS SP8 confocal laser scanning microscope and Olympus VS110 Slide scanner. The *Z*-stacks of confocal images were analyzed with ImageJ (NIH). Imaris software (Bitplane) was used for 3D rendering of confocal image *Z*-stacks. The lesion region of interest was determined by loss of MBP staining or as a hypercellular area. A similar field was used for normal-appearing white matter.

### Scanning electron microscopy.

Spinal cord samples from the lower thoracic region were embedded in 4% agarose diluted in phosphate buffer (PB, 100 mM, pH 7.4) and sectioned coronally with a vibratome (Leica Biosystems, VT1200S) to a thickness of 50 μm. Sections were examined with 1% toluidine blue diluted in double-distilled water to identify the lesion and were selected for scanning electron microscopy (SEM) processing. Sections were washed with PB, then incubated for 1 hour in a solution comprising equal volumes of 4% osmium tetroxide (Electron Microscopy Sciences) and 3% potassium ferrocyanide (BioShop) diluted in PB. After washes in double-distilled water, the sections were incubated in a filtered 1% thiocarbohydrazide solution (Electron Microscopy Sciences) for 20 minutes. After washes in double-distilled water, sections were incubated in 2% aqueous osmium tetroxide for 30 minutes. The sections were then dehydrated in increasing concentrations of ethanol for 10 minutes each (twice in 35%, once each in 50%, 70%, 80%, and 90%, and 3 times in 100%) and washed in propylene oxide (MilliporeSigma) for 10 minutes 3 times. The sections were embedded overnight in Durcupan resin (20 g component A, 20 g component B, 0.6 g component C, 0.4 g component D; MilliporeSigma) and delicately placed for flat embedding on fluoropolymer films (ACLAR, Electron Microscopy Sciences). After resin polymerization, the region of interest was dissected from the embedded sections and glued onto resin blocks for ultramicrotomy using a Leica ARTOS 3D. Sections from 2–4 levels per animal (9–10 μm apart) were cut at a thickness of 73 nm and placed onto silicon wafers for SEM imaging using a Zeiss Crossbeam 350 SEM operating at a voltage of 1.4 kV and 1.2 nA current. Images of the ultrathin sections were first acquired at a resolution of 100 nm per pixel to allow for localization of the targeted lesion and its borders. The borders of the lesion were next imaged at a resolution of 5 nm per pixel for ultrastructural analyses. Images were stitched and exported as TIFs using the software Zeiss Atlas 5 (Fibics).

Images were analyzed using QuPath (v0.3.2) and ImageJ (v1.53a, NIH) software. To quantify g-ratio, the “polygon tool” in QuPath was used to trace the perimeter (micrometers) of axons, inner myelin border, and outer myelin border. Traced regions were then exported to ImageJ, where the Feret Diameter plug-in was used to calculate the diameter of each aspect of the myelinated axon. A total of 150 axons per region of interest for each animal were included to calculate g-ratio.

### Spinal cord cell isolation for scRNA-Seq.

EAE mice from the WT, *Fbln2^+/–^*, and *Fbln2^–/–^* groups (3 mice in each group) were euthanized with a lethal dose of ketamine and xylazine 18 days after EAE induction, and then perfused via cardiac puncture with 15 mL of HBSS (without Ca^2+^, Mg^2+^; Thermo Fisher Scientific) containing 1 μM flavopiridol (MilliporeSigma) and 5 μg/mL actinomycin D (MilliporeSigma). Spinal cords were isolated and kept in 1 mL of dissection buffer containing HBSS (without Ca^2+^, Mg^2+^), 1 μM flavopiridol, 5 μg/mL actinomycin D, and 27.1 μg/mL anisomycin (MilliporeSigma). Thoracic and lumbar parts of spinal cords were cut into pieces. The single-cell suspension was prepared using a Neural Tissue Dissociation kit (Miltenyi Biotec) and gentleMACS Dissociator with Heaters (Miltenyi Biotec) according to the manufacturer’s instructions. Transcription and translation inhibitors (1 μM flavopiridol, 5 μg/mL actinomycin D, and 27.1 μg/mL anisomycin) were added to enzyme mix. Myelin debris was removed from the cell suspension using Debris Removal solution (Miltenyi Biotec). Cells were then resuspended in PBS containing 1% heat-inactivated FBS (MilliporeSigma).

### scRNA library preparation.

Once a single-cell suspension was generated as described above, scRNA-Seq was performed using the 10x Genomics platform with Chromium Next GEM Single Cell 3′ reagent kits v3.1. An appropriate volume of cells, as determined from the user guide for recovery of 4,000 cells, was loaded on the Chromium single-cell controller chip. The Chromium Next GEM Single Cell 3′ v3.1 library and gel bead kit were used to prepare scRNA-Seq libraries. Post–cDNA amplification quality control and quantification, as well as library construction quality control, were done with an Agilent Bioanalyzer high-sensitivity DNA chip for use with the Agilent 4200 TapeStation System. For sequencing, all 9 libraries were pooled and loaded at 300 pM on an Illumina NovaSeq 6000 sequencing system using an S1 flow cell. A 28 bp read 1 was used to sequence the cell barcode and unique molecular identifier.

Ten–base pair i5 and i7 index reads were used to sequence the sample index, and a 90 bp read 2 was used to sequence the transcript using paired-end, dual-index sequencing.

### scRNA-Seq analysis.

The base call files were processed using 10x Genomics Cell Ranger v6.1 pipeline with reads aligned to the mm10 mouse reference transcriptome. The cellranger aggregate pipeline was run to generate an expression matrix with the 9 combined libraries, with the normalization setting set to “None.” The sequencing depth obtained ranged from 38,700 to 48,959 reads per cell. The generated expression matrix was then analyzed using the package Seurat v3 in R v4.2.3 (45). The expression matrix comprised 36,251 cells. The data were filtered for the following parameters: cells with more than 200 and fewer than 7,500 genes and percentage of mitochondrial genes less than 10%. After filtering, the expression matrix contained 34,395 cells. Data from all 9 libraries were then integrated and normalized with the SCTransform function in Seurat using all 23,312 features, 3,000 variable features. Cell recovery from all 3 groups was comparable (WT, 12,606; Het, 11,073; Homo, 10,716 cells). A principal component analysis (PCA) reduction was performed, and 30 significant PCA dimensions were taken into account. Clusters were determined using FindNeighbors and FindClusters function, which was performed with a resolution of 0.3. Unsupervised integrated alignment of all cells from the 9 spinal cords of EAE mice delineated 17 clusters that were present in all experimental groups (Figure 2F). Using lineage cell markers, cluster 0 was identified as border-associated macrophages based on various markers (*Arg1*, *Lyz2*, *Ms4a7*, *Lgals3*, *Fabp5*); clusters 1 and 6 as homeostatic microglia (*Cx3cr1*, *Tmem119*, *P2ry12*, *Siglech*); cluster 2 as activated microglia (*C1qa*, *Cst3*, *Trem2*, *Ctsz*, *Hexb*); cluster 3 as T cells (*Skap1*, *Cd3g*, *Trbc2*, *Cd4*, *Cd8a*); clusters 4 and 12 as oligodendrocyte-lineage cells (*Plp1*, *Ptgds*, *Nkain2*, *Apod*, *Olig1*, *Mbp*, *Mog*); cluster 5 as activated peripheral monocytes/macrophages (*Ccr2*, *Msr1*, *Mrc1*, *Ms4a7*, *Ly6c*); cluster 7 as dendritic cells (*H2-Ab1*, *H2-Aa*, *Cd74*, *Napsa*, *Ciita*, *Alcam*); cluster 8 as NK/NK T cells (*Gzma*, *Klre1*, *Nkg7*, *Cd8a*, *Cd3e*, *Skap1*, *Trbc2*); cluster 9 as neutrophils (*Ly6g*, *Retnlg*); cluster 10 as B cells (*Igkc*, *Cd79b*, *Cd19*, *Ighm*); cluster 11 as proliferating cells (*His1h1b*, *Top2a*, *Birc5*, *Pclaf*); cluster 13 as fibroblasts (*Mgp*, *Sparcl1*, *Col1a2*, *Pdgfrb*, *Aqp4*, *Slc4a4*, *Igfbp7*); and cluster 14 as astrocytes (*Nnat*, *Ecrg4*, *Mt3*, *Aqp4*, *Gfap*, *Sparcl1*, *Slc1a2*) (Figure 2, E and F; Supplemental Figure 2, A and B; and Supplemental Table 1). Oligodendrocyte-lineage cells (2,592 cells) were subset and reclustered into 7 distinct subclusters determined by 10 principal components and 0.4 clustering resolution (Figure 3A; Supplemental Figure 2, C and D; and Supplemental Table 3). Differentially expressed genes (DEGs) were determined through the FindMarkers functions. DEGs with a statistically significant *P* value less than 0.05 were included for analysis.

### Sequencing and analysis of total RNA for OPCs treated with or without FBLN2.

Three hundred thousand OPCs were grown on FBLN2-coated (10 μg/mL) or PBS-coated (control) wells for 6 hours in differentiation medium (3 wells per condition). RNA was isolated from cells using the RNeasy Kit (QIAGEN) with DNA removal and according to the manufacturer’s instructions. Quality and quantity of the isolated RNA were determined by TapeStation and Fragment Analyzer (Agilent Technologies). RNA was subjected to enrichment of polyadenylated [poly(A)] mRNA and cDNA synthesis, and then prepared into libraries using NEBNext Ultra II Directional RNA Library Prep Kit (New England Biolabs). Libraries were then sequenced with a NextSeq 500 system (Illumina) using a single NextSeq 75 cycle high-output run. FastQC (v0.11.5) was used to determine the quality of sequencing reads, and cutAdapt (v1.13) was used for quality trimming (q20). Kallisto (v0.43.1) was used for quantifying abundances of transcripts. Kallisto index was built with reference transcriptome GRCm39. The R package tximport was used to summarize transcript level abundances from kallisto to gene level. The R package DESeq2 was used for differential expression analysis. DEGs with adjusted *P* value less than 0.05 were considered statistically significant and included for analysis.

### IPA.

DEGs from scRNA-Seq or bulk RNA-Seq experiments were analyzed using IPA software (QIAGEN). The Core Expression Analysis based on the average log fold change ratios was performed, where both direct and indirect relationships and interaction and causal networks filtered by experimentally observed confidence were predicted.

### Protein isolation and measurement of protein concentration.

Protein from spinal cord tissues or cell culture was isolated using ice-cold radioimmunoprecipitation assay (RIPA) lysis and extraction buffer (Thermo Fisher Scientific) containing protease and phosphatase inhibitors (Cell Signaling Technology). Astrocyte medium was removed, and cells left behind in each well of a 6-well plate (1 million cells) were lysed in 500 μL of cold RIPA buffer. After 5 minutes, lysate was collected using a cell scraper and then centrifuged at 15,000*g* for 15 minutes at 4°C. The supernatant containing protein was kept at –80°C. Proteins were quantified using the bicinchoninic acid assay relative to a BSA standard.

### Western blot.

The proteins were loaded into SDS gels (NuPAGE 4%–12% Bis-Tris Gel, Invitrogen) and run with HiMark Pre-stained Protein Standard (Invitrogen) at 250 V for 40 minutes. Then the proteins were transferred using electroblotting to an 0.2 μm PVDF membrane (GE Healthcare Life Science). The PVDF membrane was rinsed with Tris-buffered saline (TBS) containing 0.05% Tween-20 (TBST) and blocked with 10% mass per volume (m/v) skim powdered milk in TBS for 1 hour at room temperature. Rabbit anti–mouse cleaved caspase-9 (1:500; Invitrogen, PA5-105271), rabbit anti–mouse Hes5 (1:500; Abcam, ab25374), rabbit anti–mouse/human fibulin-2 (1:200; Invitrogen, catalog PA5-75510 and PA5-79239), and anti-NOTCH1 (NICD; 1:500; Invitrogen, PA5-99448) primary antibodies were added to 3% milk in TBS and incubated overnight at 4°C. The membrane was washed 5 times (5 minutes each) with TBST followed by incubation with secondary antibodies conjugated with horseradish peroxidase (HRP) for 1 hour at room temperature. The membrane was washed 5 times (5 minutes each) with TBST before visualization using ECL substrate (SuperSignal, Thermo Fisher Scientific) and imaging with the Bio-Rad ChemiDoc system. To probe for β-actin, the membrane was washed using Restore Western blot stripping buffer (Thermo Fisher Scientific) for 30 minutes before blocking with 5% m/v BSA in TBS for 1 hour at room temperature. The membrane was then incubated with primary HRP–anti–β-actin antibody (Abcam) in 3% m/v BSA in TBS for 1 hour at room temperature before washing (5 times, 5 minutes each). Western blots were quantified using the gel analyzer function in ImageJ. The relative amount of protein was normalized to the actin bands and compared with the control bands.

### Flow cytometry of spinal cord.

Spinal cords were dissected, homogenized, and filtered through a 100 μm cell strainer (Corning). To remove the myelin debris, cells were overlaid on 90% Percoll (MilliporeSigma). Then, 37% and 30% Percoll were layered on top of the cell suspension. After centrifugation at 300*g* for 8 minutes at 4°C with no brake, immune cells were removed from the interface between 90% and 37% Percoll. Cells were then resuspended in serum-free staining buffer (BD Biosciences). After live/dead staining (1:1,000; Invitrogen, L23105) and Fc receptor blocking using anti–mouse CD16/32 (1:100; Mouse BD Fc Block, BD Biosciences), cells were immunolabeled with BUV395–anti–mouse CD45 (1:200; BD Horizon, 564279, clone 30-F11), BUV805–anti–mouse CD11b (1:200; BD Horizon, 568345, clone M1/70), and BUV615–anti–mouse CD3 monoclonal (1:100; BD OptiBuild, 751418, clone 17A2) for 30 minutes at 4°C in the dark. Finally, cells were washed and analyzed by Cytek Biosciences Aurora spectral flow cytometer and FlowJo software version 10.10 (Tree Star).

### RNA isolation, cDNA synthesis, and real-time PCR.

Total RNA was purified from cells or lumbar spinal cords using RNeasy Mini kit (QIAGEN) according to the manufacturer’s instructions and stored at −80°C. The concentration and quality of RNA were determined by measurement of absorbance at 260/280 nm using a NanoDrop spectrophotometer (Thermo Fisher Scientific). To reduce DNA contamination, samples were treated with DNase (Ribonuclease-Free DNase Set, QIAGEN). RNA was reverse-transcribed to cDNA with 1 μg total RNA using miScript II RT Kit (QIAGEN) for mRNA expression analyses according to the manufacturer’s instructions. Transcripts were quantified by real-time quantitative reverse transcriptase PCR on the QuantStudio 6 Flex Real-Time PCR System (Thermo Fisher Scientific) using QuantiFast SYBR Green master mix (QIAGEN) and QuantiTect primers: Actb (QIAGEN, QT00095242), Gapdh (QT01658962), FBLN2 (Fbln2_2272567, SBM1028417), Notch1 (QT00156982), Bcl2 (QT00156282), and Bax1 (QT00102536), with the following cycling conditions: 95°C for 5 minutes and 40 cycles of denaturation at 95°C for 30 seconds, annealing at 60°C for 30 seconds, and extension at 72°C for 30 seconds. The relative expression levels were analyzed by 2^–ΔΔCt^ method, with expression levels normalized to β-actin and Gapdh housekeeping genes.

### Cell culture.

Mouse oligodendrocyte progenitor cells (OPCs) of over 80% purity were prepared from postnatal brains as previously described (17). OPCs were plated at a density of 1 × 10^4^ cells per well in 96-well flat-bottom black/clear plates precoated with poly-l-lysine in oligodendrocyte differentiation or proliferation medium. Oligodendrocyte differentiation medium comprised DMEM containing 2% B27 supplement (Gibco), 1% oligodendrocyte supplement cocktail (described below), 1% GlutaMAX (Gibco), 100 μM sodium pyruvate (Gibco), 1% penicillin-streptomycin (Gibco), 50 μg/mL holo-transferrin (MilliporeSigma), 5 μg/mL *N*-acetyl-l-cysteine (MilliporeSigma), 5 μg/mL insulin (MilliporeSigma), 50 ng/mL ciliary neurotrophic factor (PeproTech), 10 μg/mL biotin (MilliporeSigma), and 0.01% (vol/vol) Trace Elements B (Thermo Fisher Scientific). Oligodendrocyte supplement cocktail contained 100 mL DMEM with 1% BSA, 0.6 mg progesterone (MilliporeSigma), 161 mg putrescine (MilliporeSigma), 0.05 mg sodium selenite (MilliporeSigma), 4 mg 3,3′,5-triiodo-l-thyronine (MilliporeSigma), and 4 mg l-thyroxine (MilliporeSigma). Oligodendrocyte proliferation medium comprised DMEM containing 2% B27 supplement, 1% GlutaMAX, 100 μM sodium pyruvate, 1% penicillin-streptomycin, 5 μg/mL insulin (MilliporeSigma), 10 ng/mL FGF factor (PeproTech), and 10 ng/mL PDGF and FGF factor (PeproTech).

Plates were coated with recombinant protein or PBS for 3 hours at 37°C; FBLN2 (1, 5, and 10 μg/mL; R&D Systems), FBLN1 (10 μg/mL; R&D Systems), FBLN3 (10 μg/mL; R&D Systems), or Jagged1 (2 μg/mL; R&D Systems) was diluted in PBS. Jagged1 was used as a positive control for Notch signaling (Supplemental Figure 7, I–K). To hinder signaling pathways, OPCs were pretreated for 30 minutes with different inhibitors, including Notch signaling (SAHM1, 10 μM), ROCK (Y27632, 1 μM), MEK/ERK (PD98059, 10 μM), p38 MAPK (SB202190, 10 μM), PI3K/AKT/mTOR (urolithin, 10 μM), Wnt/β-catenin (endo-IWR1, 1 μM), Src/Syk (MNS, 10 μM), BMP4 (LDN193189, 100 nM), or Smad3 (SIS3, 10 μM) inhibitor, as well as 50 μg/mL blocking antibodies against mouse β_3_ integrin (CD61, Invitrogen), mouse β_1_ integrin (CD29, Invitrogen), or mouse β_6_ integrin (MilliporeSigma). All inhibitors were purchased from Tocris.

### Human OPCs.

Human brain tissues were obtained from patients undergoing surgical resection to treat intractable epilepsy as described previously (17). For human neuron culture, human fetal brain tissues from legal abortions were used to culture neurons (46). The use of the surgical material and fetal samples for the current study was approved by the Conjoint Health Research Ethics Board at the University of Calgary.

Mouse primary astrocytes were obtained from a mixed glial culture after removal of OPCs and microglia. These cells were passaged twice and cultured with decreasing concentrations of FBS (3% and 1%). Before treatment with cytokines, astrocytes were seeded in 6-well plates at 1 × 10^6^ cells/mL in DMEM with 1% FBS overnight. Cells were treated with 10 ng/mL of cytokines, including TNF-α, IL-1β, IFN-γ, and TGF-β. Mouse cortical neurons and microglia of over 90% purity were prepared from neonatal brains as previously described (46).

Bone marrow–derived macrophages (BMDMs) were prepared from femora and tibiae of C57BL/6 mice. Cells were cultured in complete DMEM (10%) and 10% L929 conditioned medium at 37°C, 8.5% CO_2_ in uncoated 100 mm plastic for 8–10 days. BMDMs were then seeded in 96-well plates at a density of 5 × 10^4^ cells per well in the same medium without L929 conditioned medium. After 24 hours, the medium was changed to serum-free DMEM, and cells were treated with LPS (100 ng/mL) for 24 hours. Conditioned medium was collected from microglia and BMDM culture to measure levels of TNF-α using an ELISA kit (Invitrogen), according to the manufacturer’s instructions.

### Immunofluorescence staining of cells in culture.

Cells were fixed for 15 minutes at room temperature using 4% PFA, rinsed with PBS, and then permeabilized with 0.1% Triton X-100 for 10 minutes at room temperature. Odyssey Blocking Buffer (LI-COR) was used for a blocking step for 1 hour at room temperature. Primary antibodies were diluted in blocking buffer and added to cells. Cells were incubated with primary antibodies including mouse anti–mouse/human sulfatide O4 (1:50; R&D Systems, MAB1326), rabbit anti–mouse MBP (1:200; Abcam, ab7349), rabbit anti–mouse/human Olig2 (1:200; MilliporeSigma, ab9610), mouse anti–human MAG (1:100; Abcam, Ab89780), rabbit anti–mouse/human Iba1 (1:500; Wako, 019-19741), goat anti–mouse/human GFAP (1:1,000; Novus, NB100-53809), mouse anti–human/mouse tubulin-β_3_ (1:500; BioLegend, clone Tuj1, 801202), rabbit anti–mouse cleaved caspase-3 (BioLegend, clone Asp175, 9661), chicken anti-GFP (1:500; Aveslab, GFP-1020), and rat anti-HA tag (1:500; Novus, NBP2-50416) overnight at 4°C, then washed 3 times (5 minutes each) with PBS. Cells were subsequently incubated with corresponding fluorophore-conjugated secondary antibodies (1:400; Jackson ImmunoResearch Laboratories) and DAPI (1 μg/mL; MilliporeSigma) at room temperature for 1 hour and washed 3 times (5 minutes each) with PBS. Cells were then imaged using ImageXpress (Molecular Devices). Data from the 12 images were averaged to a single data point per well and then normalized to control wells for each experiment.

### OPC proliferation analysis.

OPCs were cultured in proliferation medium supplemented with growth factors PDGFRα (10 ng/mL) and FGF (10 ng/mL) overnight. Cells were then treated with 5-ethynyl-2′-deoxyuridine (EdU; 10 μM) for 6 hours before detection of incorporated EdU by Click-iT EdU proliferation assay. After fixation and cell permeabilization, EdU staining was conducted according to the manufacturer’s instructions using the Click-iT Plus EdU Alexa Fluor 647 imaging kit (Thermo Fisher Scientific). ImageXpress Micro XLS High-Content Analysis System was used for analysis of cells.

### Live cell imaging of OPCs.

Mouse OPCs were plated at 1 × 10^4^ cells per well in 100 μL of differentiation medium into a 96-well flat-bottom plate coated with or without FBLN2 (10 μg/mL) in the presence of propidium iodide (PI; 1 μg/mL; Thermo Fisher Scientific). The cell survival and process outgrowth were monitored using a real-time cell imaging system (IncuCyte live-cell, ESSEN BioScience Inc.). Images of the cells were taken for a total of 48 hours at 30-minute intervals.

### Cell cycle analysis.

Mouse OPCs were plated at 1 × 10^5^ cells per well in 24-well flat-bottom plates coated with PBS (control) or FBLN2 (10 μg/mL). Cells were incubated in differentiation medium for 12 hours, and then detached with Accutase (STEMCELL Technologies) and fixed with PFA 4%. Cells were stained with FxCycle PI/RNase staining solution (Thermo Fisher Scientific) and subjected to flow cytometry analysis. Attune NxT flow cytometry was used for analysis of cells (Thermo Fisher Scientific). FlowJo version 10.7.2 was used to analyze the data (Tree Star).

### T cell proliferation analysis.

Naive CD3^+^ T cells were isolated from single-cell suspensions of splenocytes (6- to 8-week-old C57BL/6 mice) using the EasySep Kit (STEMCELL Technologies) by negative selection. Purified cells were seeded in 24-well plates at a density of 1 × 10^6^ cells in complete RPMI 1640 medium (Gibco). Cells were labeled with 1 μM of CFSE dye (Thermo Fisher Scientific), and FBLN2 was then added to the test group. Cells were stimulated with anti-CD3 (0.5 μg/mL) and anti-CD28 (0.2 μg/mL) antibodies (eBioscience). After 72 hours, cells were analyzed using flow cytometry (FACS Attune NxT, Thermo Fisher Scientific) to measure CFSE dilution, where a more proliferative culture would have more cycles of diluted CFSE. FlowJo version 10.7.2 was used to analyze the data (Tree Star).

### Transfection of primary mouse OPCs.

Freshly isolated OPCs were electroporated using the 4D-Nucleofector device (Lonza) and P3 Primary Cell 4D-Nucleofector Kit (Lonza) according to the manufacturer’s instructions in 20 μL–format Nucleocuvette strips. Cells were transfected with 2 *Notch1* siRNAs (200 nM each; Invitrogen) or negative control siRNA (200 nM; Invitrogen) and 0.4 μg pmaxGFP vector using P3 Primary Cell Nucleofector Solution and program CL-133. Transfection efficiency was between 45% and 60% (Supplemental Figure 7L).

### Dual luciferase reporter assay.

The dual luciferase assay was carried out according to the instructions of the Notch Pathway Reporter kit (BPS Bioscience). Briefly, HEK293 cells (3 × 10^4^ per well) were transfected with 1 μL CSL (CBF1/RBP-Jκ) luciferase reporter vector and constitutively expressing Renilla luciferase vector (positive control) using Lipofectamine 2000 (Invitrogen). Luciferase reporter vector contains the firefly luciferase gene under control of multimerized CSL responsive element. Activity of Notch signaling results in proteolytic cleavage of the Notch receptor, releasing the active intracellular domain of NICD, which goes to the nucleus and interacts with the transcription factor CSL to activate transcription of Notch-responsive genes and luciferase (Figure 6I). After transfection (12–16 hours), medium was changed, and HEK293 cells were cultured in the presence or absence of FBLN2. After 24 hours of cultivation, luciferase activity was measured using a Dual-Luciferase Assay System (BPS Bioscience) and luminometer. The ratio of firefly luminescence to Renilla luminescence was used to normalize luciferase activity.

### Statistics.

Microsoft Excel (v2201 Build 16.0.14827.20198) was used for collating data. All graphs were generated using GraphPad Prism 9.4.0. For comparisons between 2 groups, significance was determined by unpaired 2-tailed Student’s *t* tests for parametric data and Mann-Whitney test for nonparametric data. Where multiple groups were compared, 1- or 2-way ANOVA with Bonferroni’s or Tukey’s multiple-comparison test and nonparametric Kruskal-Wallis with Dunn’s multiple-comparison test were used. EAE disease scores were analyzed with 2-way repeated-measures ANOVA with Dunnett’s multiple-comparison test. Kolmogorov-Smirnov test was applied to verify normal distribution of data. *P* values below 0.05 were considered statistically significant and are shown by the exact number or by asterisks in the figures.

### Study approval.

The use of all human tissues in this study was approved by the Conjoint Health Research Ethics Board at the University of Calgary (Ethics ID REB15-0444). All samples were collected with full written informed consent for autopsy, and their use for research was approved by the University of Montreal Hospital Research Centre research ethics committee (ethical approval BH07.001). All animal work was performed with ethics approval (protocols AC21-0174, AC21-0154, AC21-0073-4) from the Animal Care Committee at the University of Calgary under regulations of the Canadian Council of Animal Care.

### Data availability.

Raw single-cell RNA-sequencing and bulk RNA-sequencing data are available at the NCBI’s Sequence Read Archive with the BioProject accession numbers PRJNA109264 and PRJNA1106007, respectively. A Supporting Data Values file is provided as supplemental material.

## Author contributions

SG designed the project and performed the majority of experiments, analyzed the results, and wrote the first draft of the paper. CL performed some of the staining, imaging, and Western blot experiments. BML performed lysolecithin surgeries. DM helped with coronal sectioning and tissue processing. CD was critical for performing the scRNA-Seq experiments and processing bulk sequencing data. YD provided support for neuron and microglia culture. FV generated AAV vectors. CS helped with AAV injections. HL and MX contributed to the ICH data. Electron microscopy and subsequent data analysis were performed by MK, CJM, and MÈT. VWY supervised the study, provided operational support, and edited and finalized the manuscript. All authors reviewed and edited the manuscript.

## Supplementary Material

Supplemental data

Unedited blot and gel images

Supplemental tables 1-3

Supporting data values

## Version 1 05/14/2024

In-Press Preview

## Version 2 07/01/2024

Electronic publication
