# Supplementary material for: Fibulin-2 is an extracellular matrix inhibitor of oligodendrocytes relevant to multiple sclerosis
Source: J Clin Invest. 2024 May 14;134(13):e176910. doi: 10.1172/JCI176910 (PMC11213512; doi:10.1172/JCI176910)
Supplement: Supplemental data [file jci-134-176910-s079.pdf]

## Supplemental Figure 1

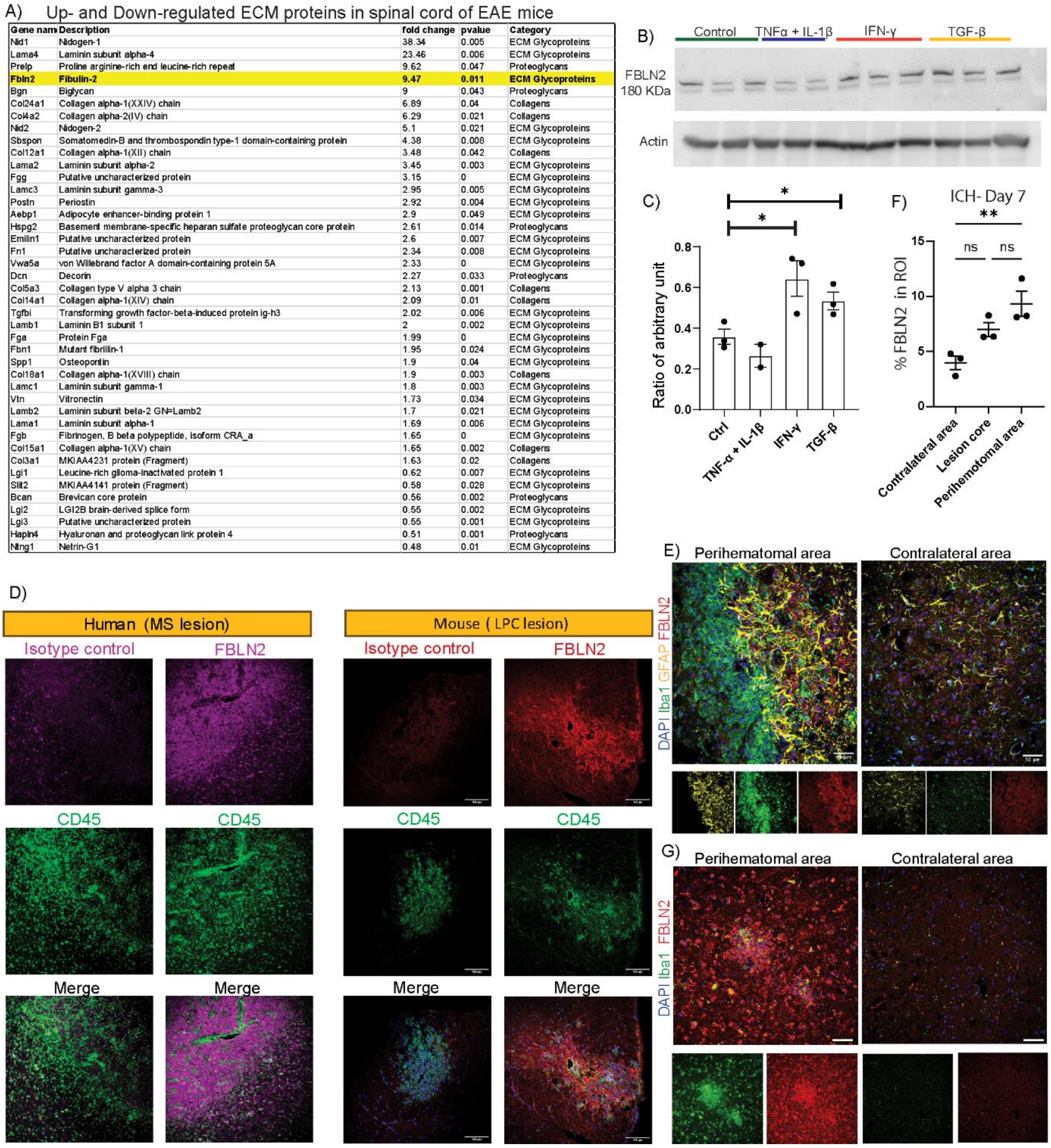

A) Up- and Down-regulated ECM proteins in spinal cord of EAE mice

| Gene nam | Description | fold change | pvalue | Category |
|---|---|---|---|---|
| Nid1 | Nidogen-1 | 38.34 | 0.005 | ECM Glycoproteins |
| Lama4 | Laminin subunit alpha-4 | 23.46 | 0.006 | ECM Glycoproteins |
| Prelp | Proline arginine-rich end leucine-rich repeat | 9.62 | 0.047 | Proteoglycans |
| **Fbln2** | **Fibulin-2** | **9.47** | **0.011** | **ECM Glycoproteins** |
| Bgn | Biglycan | 9 | 0.043 | Proteoglycans |
| Col24a1 | Collagen alpha-1(XXIV) chain | 6.89 | 0.04 | Collagens |
| Col4a2 | Collagen alpha-2(IV) chain | 6.29 | 0.021 | Collagens |
| Nid2 | Nidogen-2 | 5.1 | 0.021 | ECM Glycoproteins |
| Sbspon | Somatomedin-B and thrombospondin type-1 domain-containing protein | 4.38 | 0.008 | ECM Glycoproteins |
| Col12a1 | Collagen alpha-1(XII) chain | 3.48 | 0.042 | Collagens |
| Lama2 | Laminin subunit alpha-2 | 3.45 | 0.003 | ECM Glycoproteins |
| Fgg | Putative uncharacterized protein | 3.15 | 0 | ECM Glycoproteins |
| Lamc3 | Laminin subunit gamma-3 | 2.95 | 0.005 | ECM Glycoproteins |
| Postn | Periostin | 2.92 | 0.004 | ECM Glycoproteins |
| Aebp1 | Adipocyte enhancer-binding protein 1 | 2.9 | 0.049 | ECM Glycoproteins |
| Hspg2 | Basement membrane-specific heparan sulfate proteoglycan core protein | 2.61 | 0.014 | Proteoglycans |
| Emilin1 | Putative uncharacterized protein | 2.6 | 0.007 | ECM Glycoproteins |
| Fn1 | Putative uncharacterized protein | 2.34 | 0.008 | ECM Glycoproteins |
| Vwa5a | von Willebrand factor A domain-containing protein 5A | 2.33 | 0 | ECM Glycoproteins |
| Dcn | Decorin | 2.27 | 0.033 | Proteoglycans |
| Col5a3 | Collagen type V alpha 3 chain | 2.13 | 0.001 | Collagens |
| Col14a1 | Collagen alpha-1(XIV) chain | 2.09 | 0.01 | Collagens |
| Tgfbi | Transforming growth factor-beta-induced protein ig-h3 | 2.02 | 0.006 | ECM Glycoproteins |
| Lamb1 | Laminin B1 subunit 1 | 2 | 0.002 | ECM Glycoproteins |
| Fga | Protein Fga | 1.99 | 0 | ECM Glycoproteins |
| Fbn1 | Mutant fibrillin-1 | 1.95 | 0.024 | ECM Glycoproteins |
| Spp1 | Osteopontin | 1.9 | 0.04 | ECM Glycoproteins |
| Col18a1 | Collagen alpha-1(XVIII) chain | 1.9 | 0.003 | Collagens |
| Lamc1 | Laminin subunit gamma-1 | 1.8 | 0.003 | ECM Glycoproteins |
| Vtn | Vitronectin | 1.73 | 0.034 | ECM Glycoproteins |
| Lamb2 | Laminin subunit beta-2 GN=Lamb2 | 1.7 | 0.021 | ECM Glycoproteins |
| Lama1 | Laminin subunit alpha-1 | 1.69 | 0.006 | ECM Glycoproteins |
| Fgb | Fibrinogen, B beta polypeptide, isoform CRA_a | 1.65 | 0 | ECM Glycoproteins |
| Col15a1 | Collagen alpha-1(XV) chain | 1.65 | 0.002 | Collagens |
| Col3a1 | MKIAA4231 protein (Fragment) | 1.63 | 0.02 | Collagens |
| Lgi1 | Leucine-rich glioma-inactivated protein 1 | 0.62 | 0.007 | ECM Glycoproteins |
| Slit2 | MKIAA4141 protein (Fragment) | 0.58 | 0.028 | ECM Glycoproteins |
| Bcan | Brevican core protein | 0.56 | 0.002 | Proteoglycans |
| Lgi2 | LGI2B brain-derived splice form | 0.55 | 0.002 | ECM Glycoproteins |
| Lgi3 | Putative uncharacterized protein | 0.55 | 0.001 | ECM Glycoproteins |
| Hapln4 | Hyaluronan and proteoglycan link protein 4 | 0.51 | 0.001 | Proteoglycans |
| Ntng1 | Netrin-G1 | 0.48 | 0.01 | ECM Glycoproteins |



**Supplemental Figure 1. FBLN2 is upregulated in activated astrocytes in culture and intracerebral hemorrhage (ICH) injury**.

(**A**) Up- and down-regulated ECM proteins in spinal cord of EAE mice identified from a proteomic study[19]. (**B**) Western blot analysis of FBLN2 in mouse astrocytes cultured with or without indicated treatments for 24 h. (**C**) Bar graph comparing the signal ratio of FBLN2 to β-actin in untreated astrocyte (control) to TNF-α+IL1-β (10 ng/mL each), IFN-γ (10 ng/mL) and TGF-β (10 ng/mL) treated cells. *n*= 3 for control, IFN-γ and TGF-β groups, 2 for TNF-α+IL1-β group. (**D**) Representative confocal images of human MS brain lesion (left) and LPC-induced lesion defined by accumulation of CD45+ immune cells stained for fibulin-2 or isotype antibody control. Scale bar, 100 µm. (**E**) Representative confocal images and (**F**) FBLN2 percent area of perihematomal (lesion core is at the bottom left) and contralateral hemisphere (uninjured) region of the mouse brain 7 days after collagenase-induced ICH (*n* = 3 mice). (**G**) Representative confocal images of human brain 3 days after hemorrhagic stroke comparing perihematomal and contralateral hemisphere (no evidence of pathology) areas. Scale bar, 50 µm. Data are presented as Mean ± SEM from one representative experiment. Experiments were repeated twice; One-way ANOVA - Bonferroni post hoc; *$p < 0.05$, **$p < 0.01$.

## Supplemental Figure 2

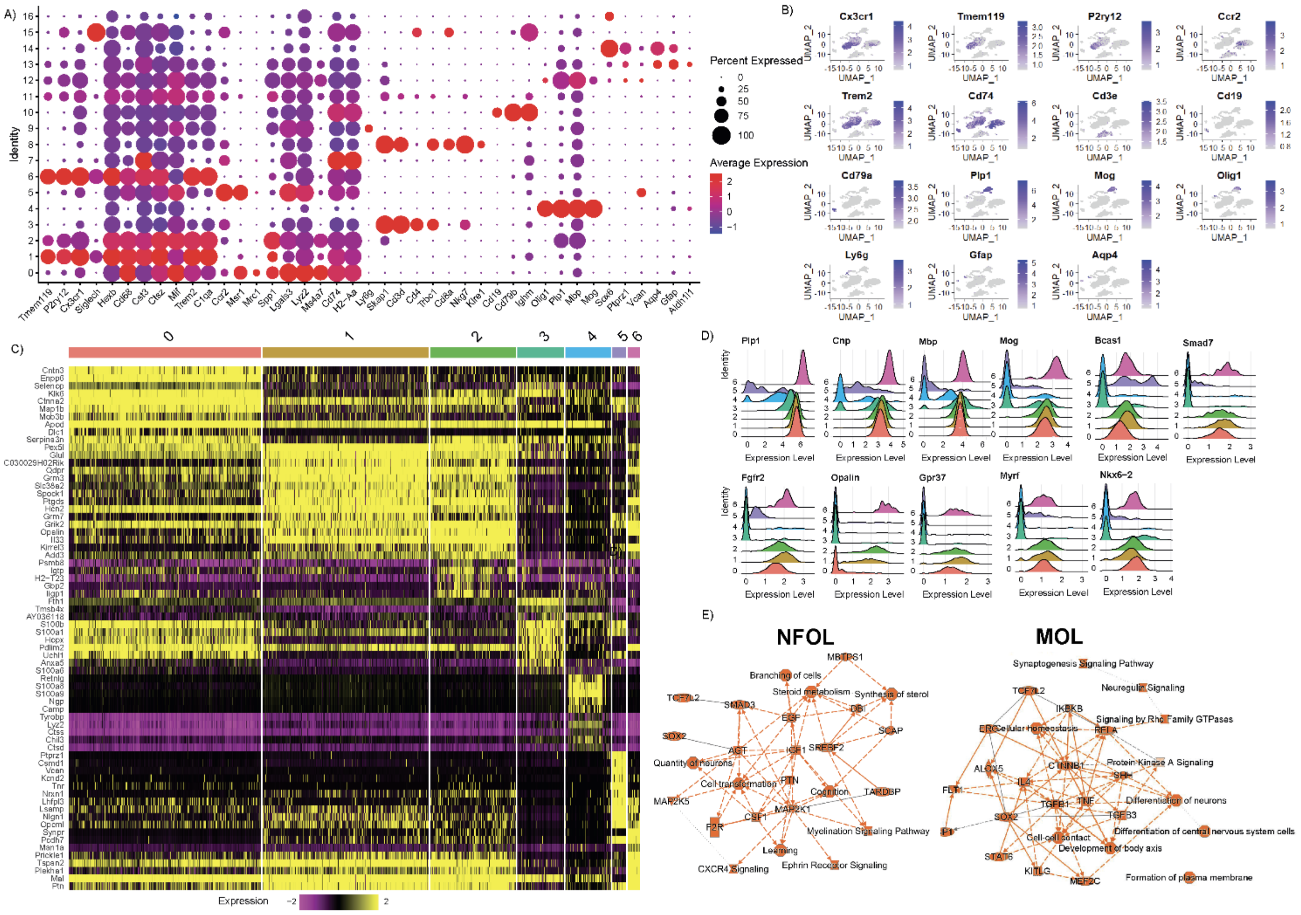


**Supplemental Figure 2. Additional scRNA-seq analysis**.

(**A**) Dotplot showing the expression of lineage markers for homeostatic microglia, activated microglia or macrophages, BAM, DC, neutrophils, T cells, NKT cells, B cells, oligodendrocyte lineage cells and astrocytes across the clusters. The size of the dot corresponds to the percentage of cells expressing the gene in each cluster. The color represents the average gene expression level. (**B**) Feature UMAP plot showing the distribution of various genes used to define clusters. Color bars indicate average expression. (**C**) Heat map of top marker genes of different subclusters of oligodendrocyte lineage cells. (**D**) Ridge plots comparing expression of select remyelination-related genes across oligodendrocyte subclusters (0: DA-MOL, 1: MOL, 2: IFN-MOL, 3 and 4: Stresses-OL, 5: OPC/COP, 6: NFOL). (**E**) Graphical summary of IPA predicted activated pathways in NFOL (subcluster 6) and MOL (subcluster 1). ScRNA-seq data in each experimental group acquired from 3 mice.

## Supplemental Figure 3

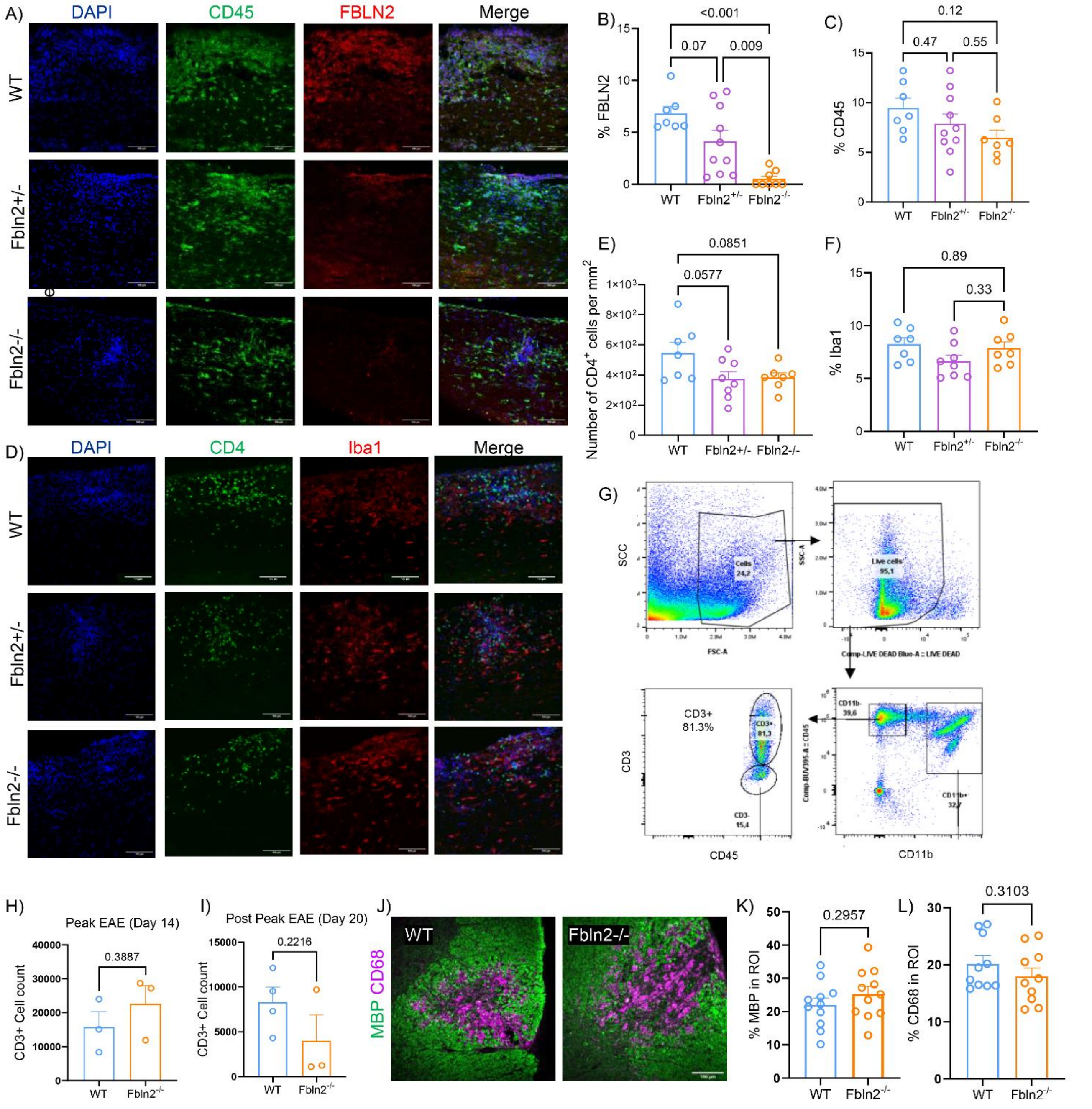


**Supplemental Figure 3. FBLN2 deficiency does not affect the extent of inflammation.**

(**A**) Representative images of longitudinal spinal cord sections from EAE WT, *Fbln2+/-* and *Fbln2-/-* mice. Tissues were stained for CD45 and FBLN2. DAPI was used to label nuclei. (**B** and **C**) Bar graphs comparing the percent of ROI that is FBLN2$^{+}$ (B) or CD45$^{+}$ (C). (**D-F**) Representative images of EAE lesion from WT, *Fbln2+/-* and *Fbln2-/-* mice (D) and quantifications for number of CD4 per mm2 of EAE lesions (E) and Iba1 percent area of the lesion (F) (*n* = 7-10 mice; One-way ANOVA - Bonferroni post hoc). (**G-I**) Flow cytometry plots showing the gating strategies (G) and bar graphs comparing number of CD3$^{+}$ T cells at peak (H) and post peak EAE (I) in the spinal cord of WT and *Fbln2-/-* mic (*n*= 3 mice; two-tailed unpaired Student's t test). **(J)** Representative images of coronal sections of LPC lesions 14 dpi comparing WT and homozygous *Fbln2*$^{-/-}$ mice. Tissues were labeled with MBP and CD68. (**K** and **L**) Bar graphs showing the percent of MBP (K) and CD68 (L) area within the lesion *(n*= 10 mice; two-tailed unpaired Student's t test). Data are presented as Mean ± SEM. All images were acquired by immunofluorescent laser confocal microscope (Z-stack). Scale bars, 100 μm.

**Supplemental Figure 4**

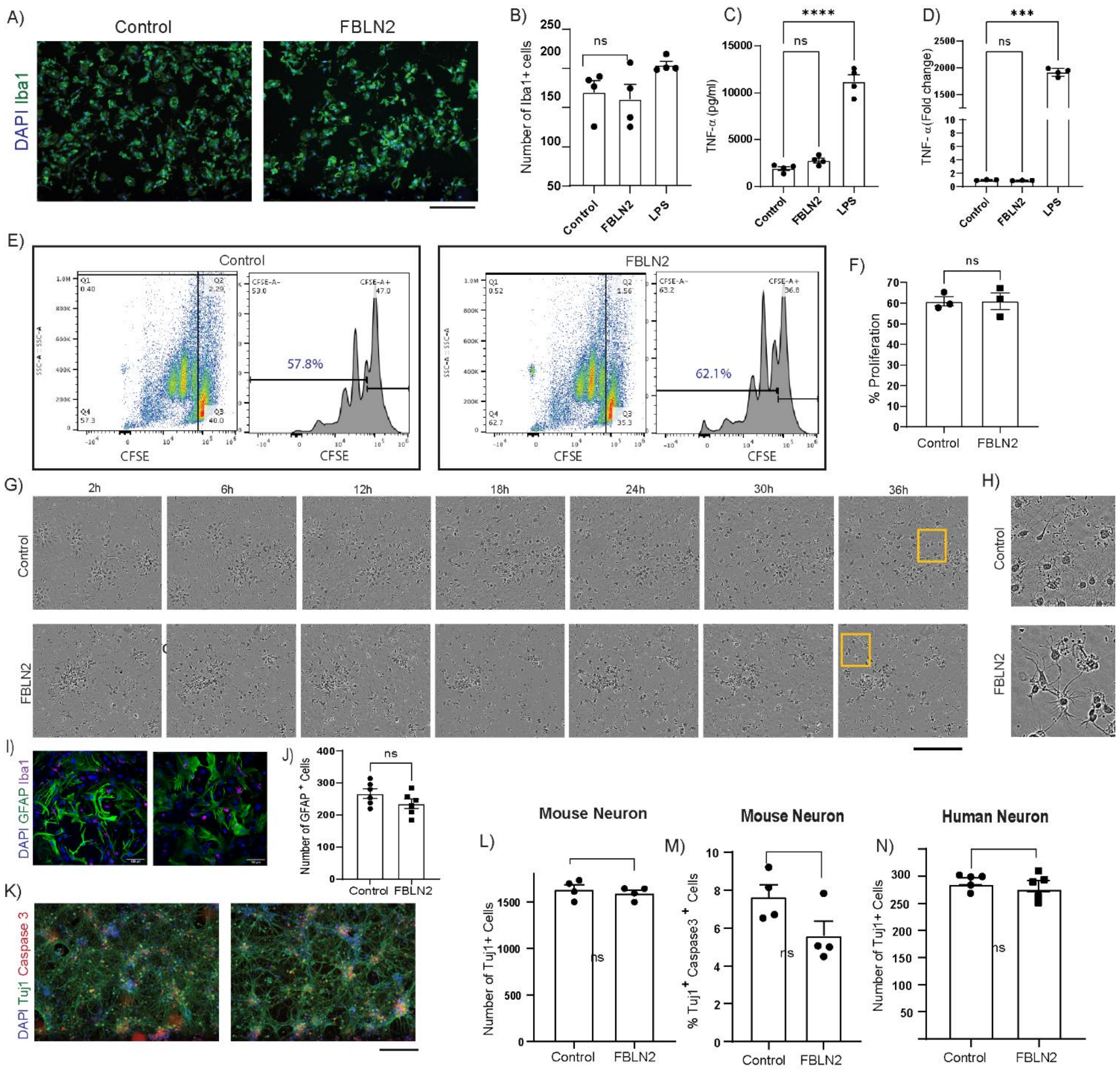

**Supplemental Figure 4. FBLN2 does not affect microglia/macrophages, lymphocytes astrocytes and neurons.**

(**A**) Representative images of primary mouse microglia labeled with Iba1 and DAPI after 24h of culture on PBS (control) or FBLN2 (10 µg/ml) coated wells. (**B**) Quantification comparing number of DAPI+Iba1+ microglia in the presence and absence of FBLN2 after 24h. (**C** and **D**) Level of TNF-α measured by ELISA in the conditioned medium of mouse microglia (C) and bone marrow derived macrophages (D) treated with FBLN2 or LPS for 24h. *n*= 4 replicates; One-way ANOVA - Bonferroni post hoc; ***p < 0.001. (**E**) Flow cytometry CFSE plots showing T cell proliferation after 3 days in the presence or absence of FBLN2 (10 µg/ml) where the peaks to the left represent cycles of cell division. (**F**) Quantification comparing the percentage of dividing cells (*n*= 3 replicates, two-tailed unpaired Student's t test). (**G** and **H**) Representative bright-field microscopy images from live imaging of mouse OPC plated on PBS (control) and FBLN2 (10 µg/ml) coated wells at different time points. Yellow boxes specify the areas shown at higher magnification in H. (**I**) Representative images of primary mouse astrocytes stained for GFAP, Iba1 and DAPI cultured on PBS (control) or FBLN2 coated (10 µg/ml) wells. (**J**) Bar graph comparing the number of DAPI+GFAP+ astrocytes after 72h (*n*= 6 replicates; two-tailed unpaired Student's t test). (**K**) Representative images of primary mouse neurons labeled with β-tubulin (Tuj1) and caspase3 after 24h of culture on PBS

(control) or FBLN2 coated (10 µg/ml) wells. DAPI was used to label nuclei. (**L**-**N**) Bar graph comparing the number of DAPI+Tuj1+ (L), percentage of Tuj1+ Caspase3+ primary mouse neurons (M) and number of DAPI+Tuj1+ primary human neurons (N) after 24h (*n*= 4-5 replicates; two-tailed unpaired Student's t test). Data are presented as Mean ± SEM from one representative experiment. Experiments were repeated twice. Scale bar, 100 µm.

## Supplemental Figure 5

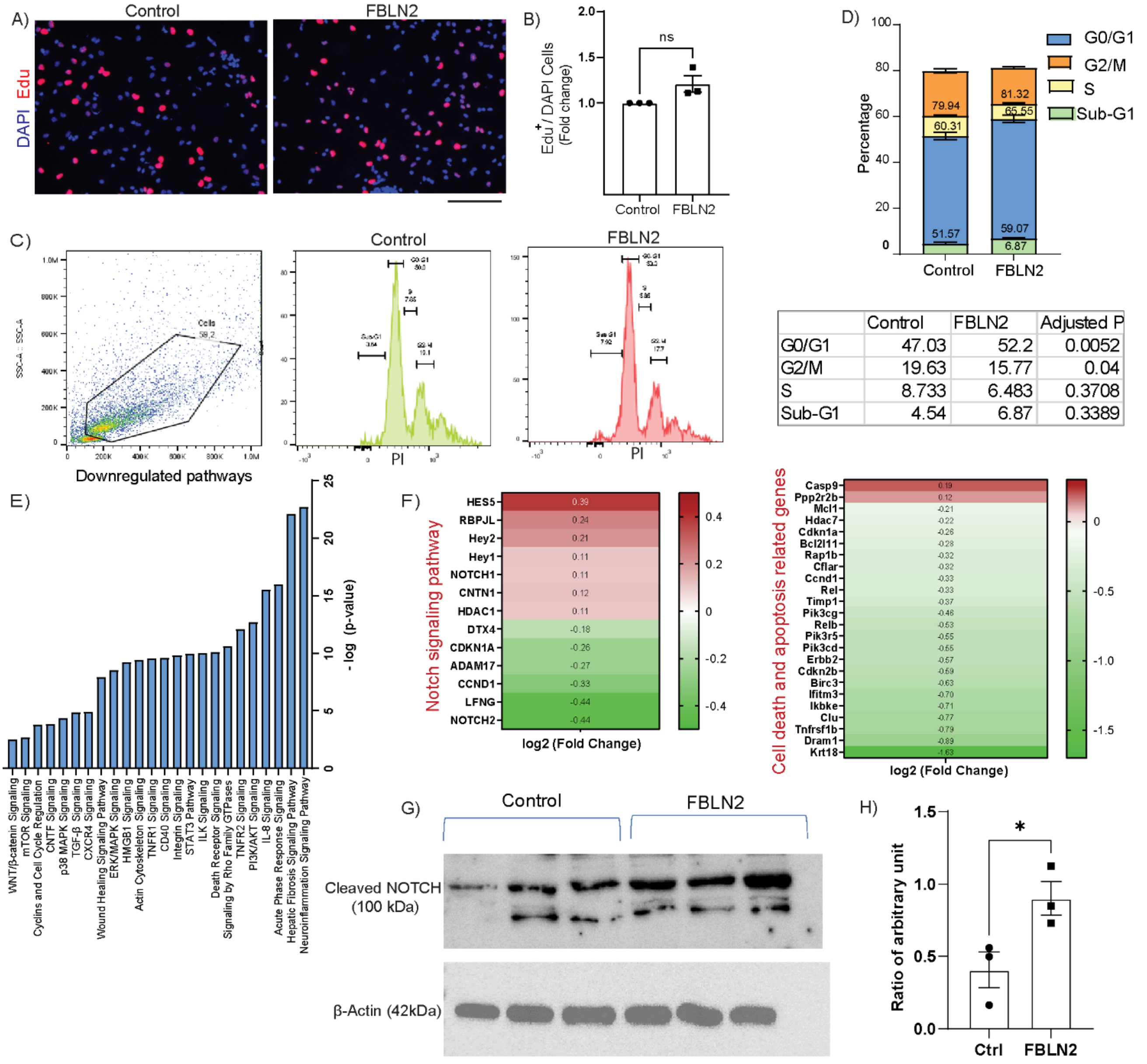


| | Control | FBLN2 | Adjusted P |
|---|---|---|---|
| G0/G1 | 47.03 | 52.2 | 0.0052 |
| G2/M | 19.63 | 15.77 | 0.04 |
| S | 8.733 | 6.483 | 0.3708 |
| Sub-G1 | 4.54 | 6.87 | 0.3389 |

**Supplemental Figure 5. FBLN2 does not inhibit OPC proliferation while it induces cell cycle arrest in differentiating OPCs**

(**A** and **B**) Representative images (A) and bar graph (B) comparing the proliferation of mouse OPCs in the presence or absence of FBLN2 (10 µg/ml) by measuring incorporation of EdU into DNA following 24h culture in the proliferation media (*n*= 3 replicates; two-tailed unpaired Student's t test Scale bar,100 µm.). (**C**) Cell cycle analysis using PI staining and flow cytometry of mouse OPCs cultured on control and FBLN2 (10 µg/ml) coated wells in differentiation media after 12h. (**D**) Bar graph and table depicting percentage of cells in different phases of cell cycle including sub-G1, G0/G1, S and G2/M phase (*n*= 3 replicates, two-way repeated-measures ANOVA with Sidak's post-hoc test). (**E**) Top down-regulated pathways predicted by IPA from DEGs by mouse OPCs in the presence of FBLN2. (**F**) Log2 (Fold Change) of genes associated with Notch signalling pathway (left), and cell death and apoptosis (right) by FBLN2 treated OPCs. RNA sequencing data were acquired from 3 replicates per group (control or FBLN2 (10 µg/ml)). (**G** and **H**) Western blot analysis of cleaved NOTCH (NICD) (G) and quantification (H) comparing the signal ratio of NICD to β-actin in OPCs plated on control and FBLN2 (10 µg/ml) coated wells in differentiation media for 12 h (*n*= 3 replicates; two-tailed unpaired Student's t test; *p < 0.05). Data are presented as Mean ± SEM from one representative experiment. Experiments for B and D were repeated twice.

## Supplemental Figure 6

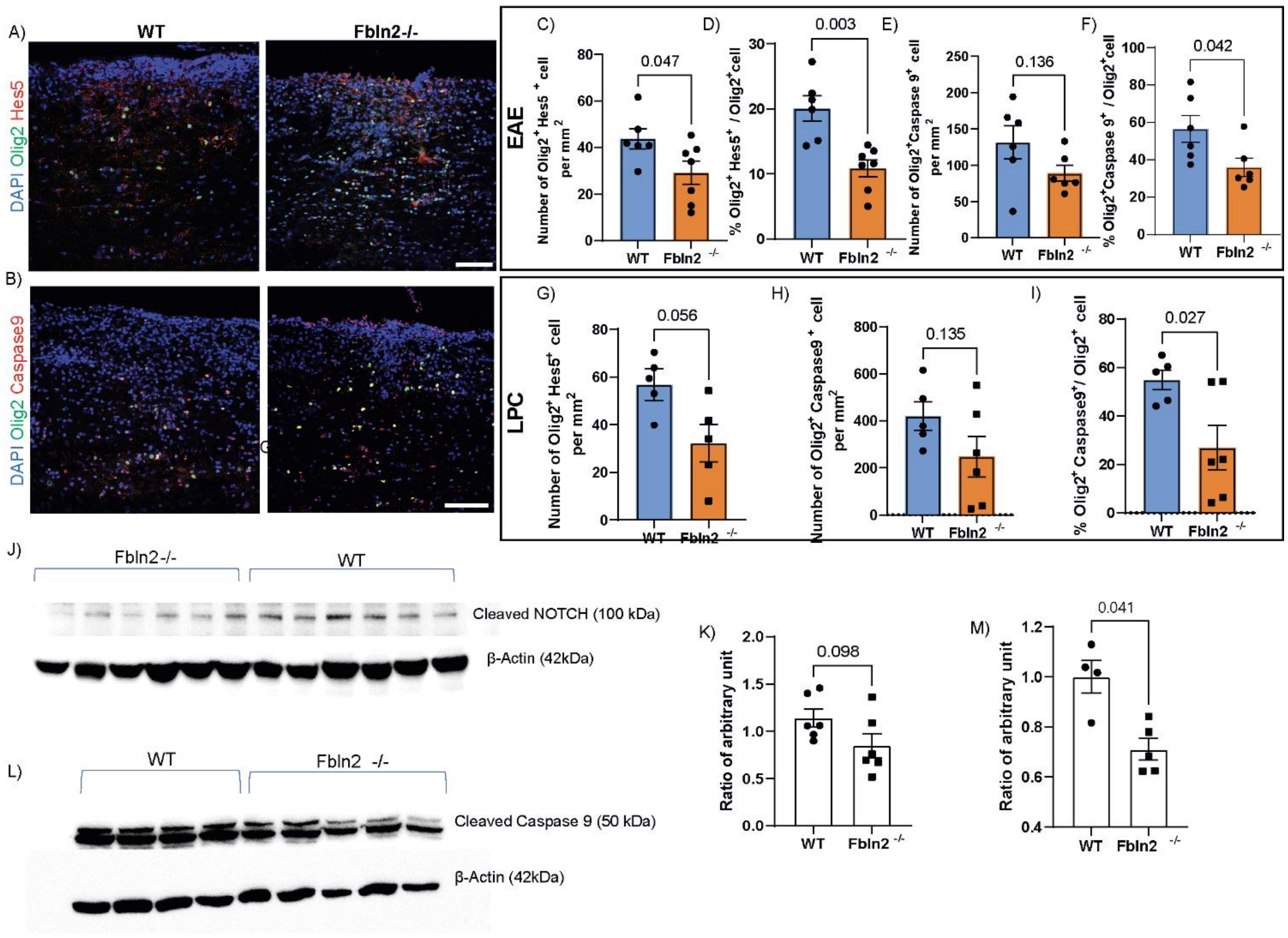


**Supplemental Figure 6. Additional data from FBLN2 knockout mice**.

(**A** and **B)** Representative images of longitudinal sections of spinal cord from EAE mice comparing WT and *Fbln2 -/-* mice. Tissues were stained for DAPI, Olig2 and Hes5 or cleaved caspase 9. (**C-F**) Bar graphs comparing the number (C) and proportion (D) of Olig2$^+$ Hes5$^+$ oligodendrocytes, number (E) and proportion (F) of Olig2$^+$ Caspase 9$^+$ oligodendrocytes within the EAE lesions (*n*= 6 mice). (G-I) Number of Olig2$^+$ Hes5$^+$ oligodendrocytes (G), Olig2$^+$ Caspase 9$^+$ oligodendrocytes (H) per mm$^2$ and proportion of Olig2$^+$ Caspase 9$^+$ cells (I) within the ROI of LPC lesions 14 dpi (*n*= 5-6 mice). (**J**) Western blot analysis of cleaved NOTCH (NICD) and (**K**) bar graph comparing the signal ratio of NICD to β-actin between WT and *Fbln2-/-* mice (*n*= 6 mice). (**L**) Western blot analysis of cleaved caspase 9 and (**M**) quantification for signal ratio of cleaved caspase 9 to β-actin in WT and *Fbln2-/-* mice (*n*= 4-5 mice). Data are presented as Mean ± SEM; two-tailed unpaired Student's t test. Images were acquired by immunofluorescent laser confocal microscope (Z-stack). Scale bars, 100 μm.

## Supplemental Figure 7

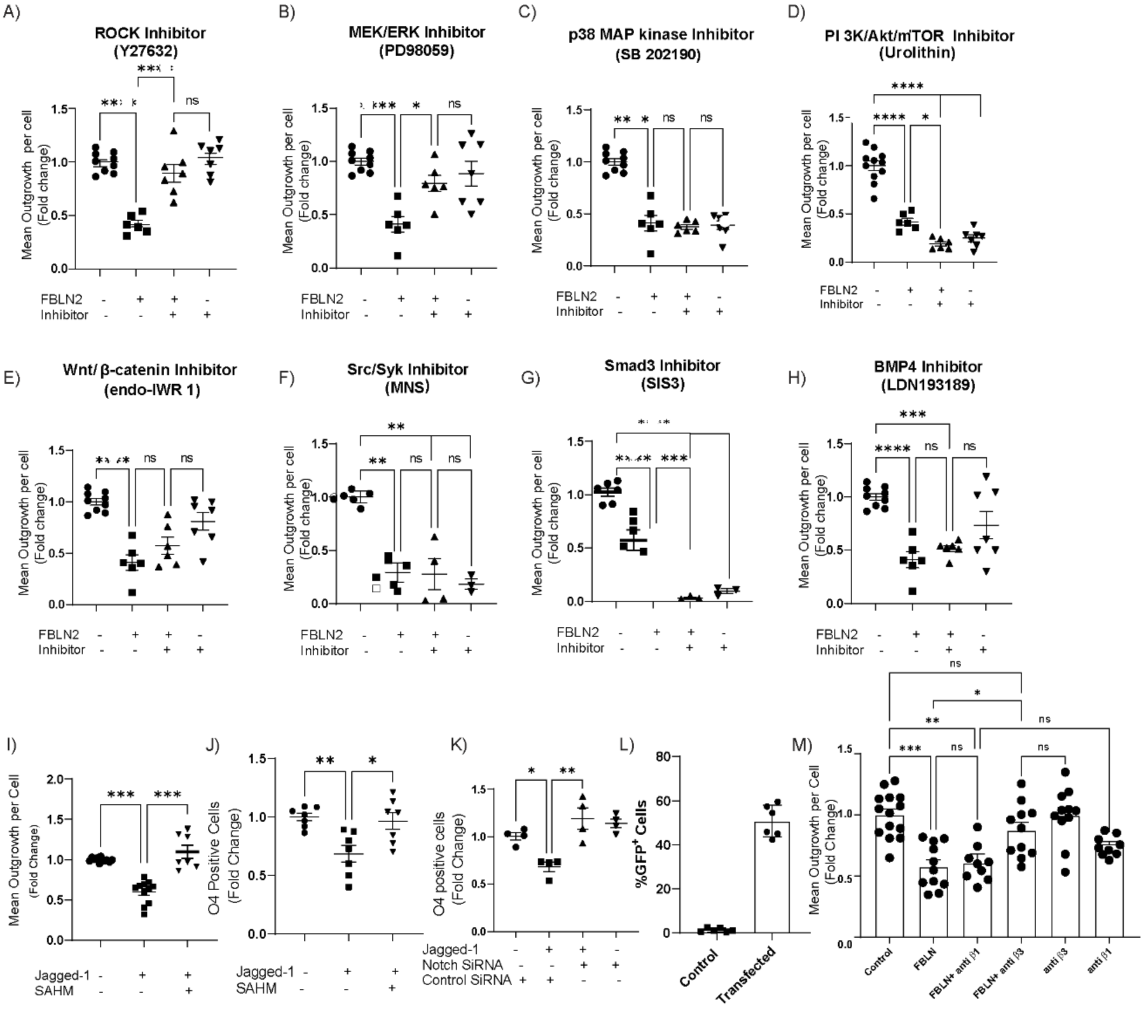


**Supplemental Figure 7. Additional OPC culture experiments.**

(**A**-**H**) Bar graphs comparing the mean process outgrowth of mouse OPCs cultured on PBS (control) and FBLN2 (10 µg/ml) coated wells with or without 1µM ROCK (A), 10µM MEK/ERK (B), 10µM P38 MAP kinase (C), 10µM PI3k/AKT/mTOR (D), 1µM Wnt/βcatenin (E), 10µM Src/Syk (F), 10µM Smad3 (G) and 100nM BMP4 (H) signaling inhibitors after 24h (*n*= 6-9 replicates from 2-3 separate experiments). (**I** and **J**) Quantification for mean process outgrowth (**I**) and number of O4$^+$ cells (J) of mouse OPCs cultured on control and Jagged1 (2µg/ml) coated wells with or without Notch inhibitor (SAHM1, 10µM) after 24h (*n*= 7 replicates over two separate experiments; each experiment included 3–4 replicates). (**K**) Number of O4$^+$ cells of mouse OPCs transfected with two NOTCH1 siRNA (200nM) cultured on control and Jagged1 (2µg/ml) coated wells (*n*= 4 replicates from one representative experiment. Experiment was repeated twice). (**L**) Percentage of GFP expressing cells showing the SiRNA transfection efficiency (~50%) (*n*= 6). (**M**) Fold change in mean process outgrowth of mouse OPCs cultured on control and FBLN2 (10 µg/ml) coated wells with or without function blocking antibodies to the integrin β1or β3 (50 µg/ml) for 24h (*n*= 9-11 replicates over three separate experiments). Data are presented as Mean ± SEM, One-way ANOVA - Tukey post hoc; **p < 0.05, **p < 0.01, ***p < 0.001*.

## Supplemental Figure 8

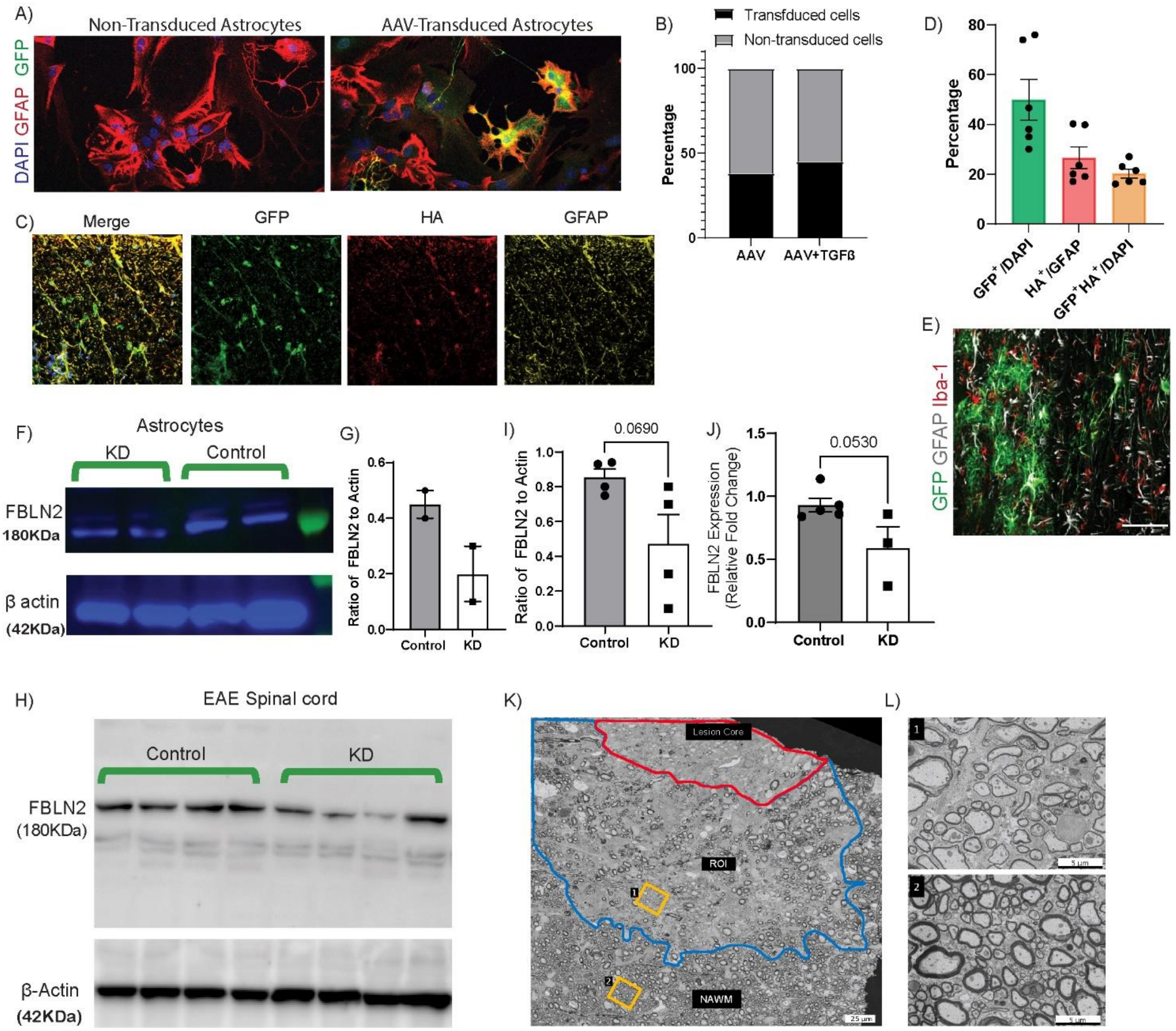


**Supplemental Figure 8. Feasibility and success of AAV-coupled CRISPR/Cas9 system to target FBLN2 in astrocytes; examples of electron micrographs**

(**A**) Representative images of mouse astrocytes stained for GFAP and GFP 48 h after transduction with GFP expressing AAV (1.5× $10^6$ vg/virus per 10000 cells). (**B**) Graph showing the percentage of GFP$^+$ cells in AAV transduced or non-transduced astrocytes with or without TGF-β (10ng/mL) treatment (*n*= 3 replicates). (**C**) Representative images of coronal sections of spinal cord from 8-week-old naive C57BL/6J mice 14 days post AAV injection (3 × $10^{11}$ vg/virus). Tissue was labeled with GFP, HA and GFAP. (**D**) Quantification showing the percentage of GFP$^+$ cells, HA$^+$ astrocytes and double positive cells for GFP and HA (*n*= 6 ROIs from three mice). (**E**) Representative image of longitudinal section of spinal cord from EAE mice (NAWM) labeled with GFP, GFAP and Iba1. (**F**) Western blot analysis of FBLN2 in mouse non-transduced and AAV-transduced cultured astrocytes for 48h. (**G**) Bar graph comparing the signal ratio of FBLN2 to β-actin in cultured mouse non-transduced and AAV-transduced astrocytes. (**H** and **I**) Western blot analysis of FBLN2 (H) and quantification (I) comparing the signal ratio of FBLN2 to β-actin in spinal cord lysate of AAV-injected EAE mice (FBLN2-targeted or non-target gRNA as control) (*n*= 4 mice). (**J**) Relative fold change of FBLN2 mRNA expression using Real time PCR in spinal cords of AAV-injected EAE mice (*n*= 4 mice). Data are presented as Mean ± SEM; two-tailed unpaired Student's t test. (**K** and **L**) Electron micrographs of LPC-induced lesions. The lesion core is delineated by the red line,

while the blue line outlines the ROI containing the area between the lesion border and core. Scale bars, 25 μm. The yellow square specifies the area shown at higher magnification in (L); Scale bars, 5 μm.
