# Supplementary material for: Fibulin-2 is an extracellular matrix inhibitor of oligodendrocytes relevant to multiple sclerosis
Source: J Clin Invest. 2024 May 14;134(13):e176910. doi: 10.1172/JCI176910 (PMC11213512; doi:10.1172/JCI176910)

Supplemental Figure 1B

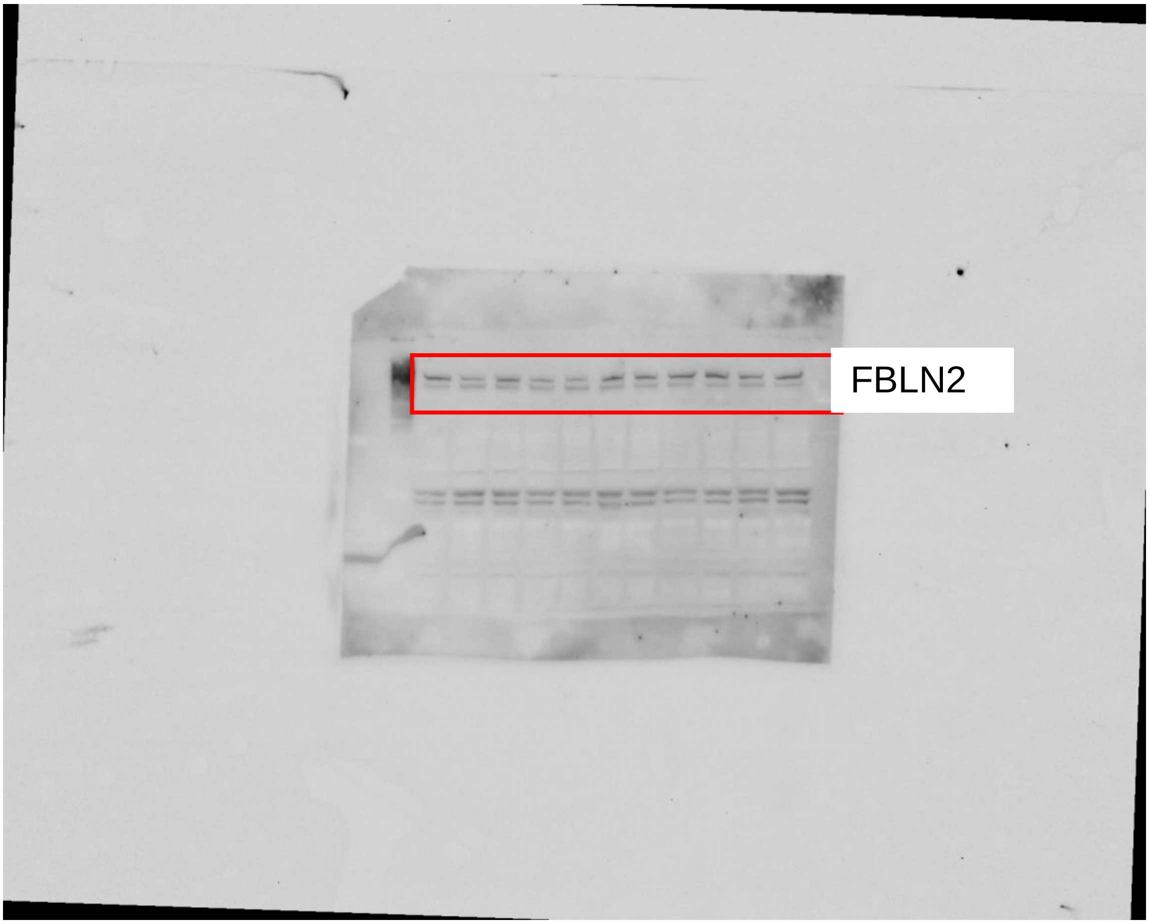

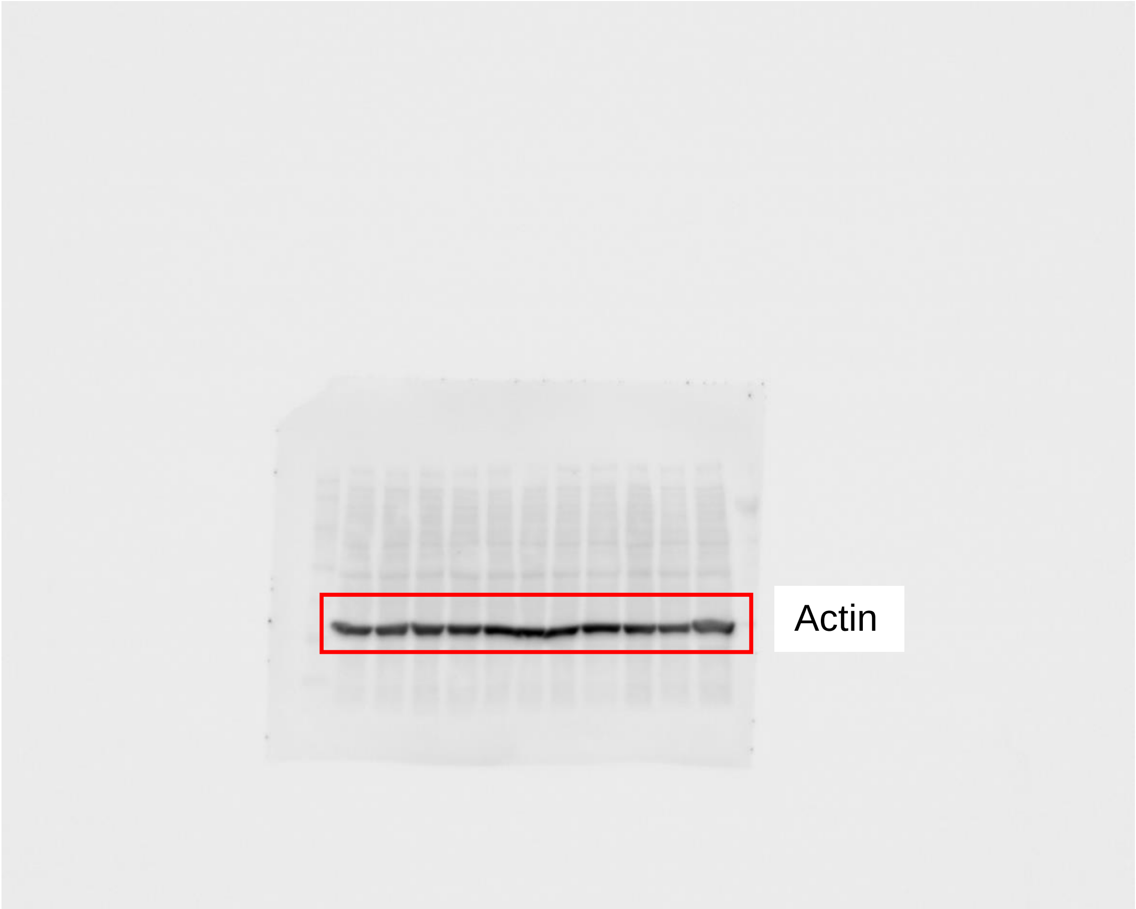

Supplemental Figure 5G

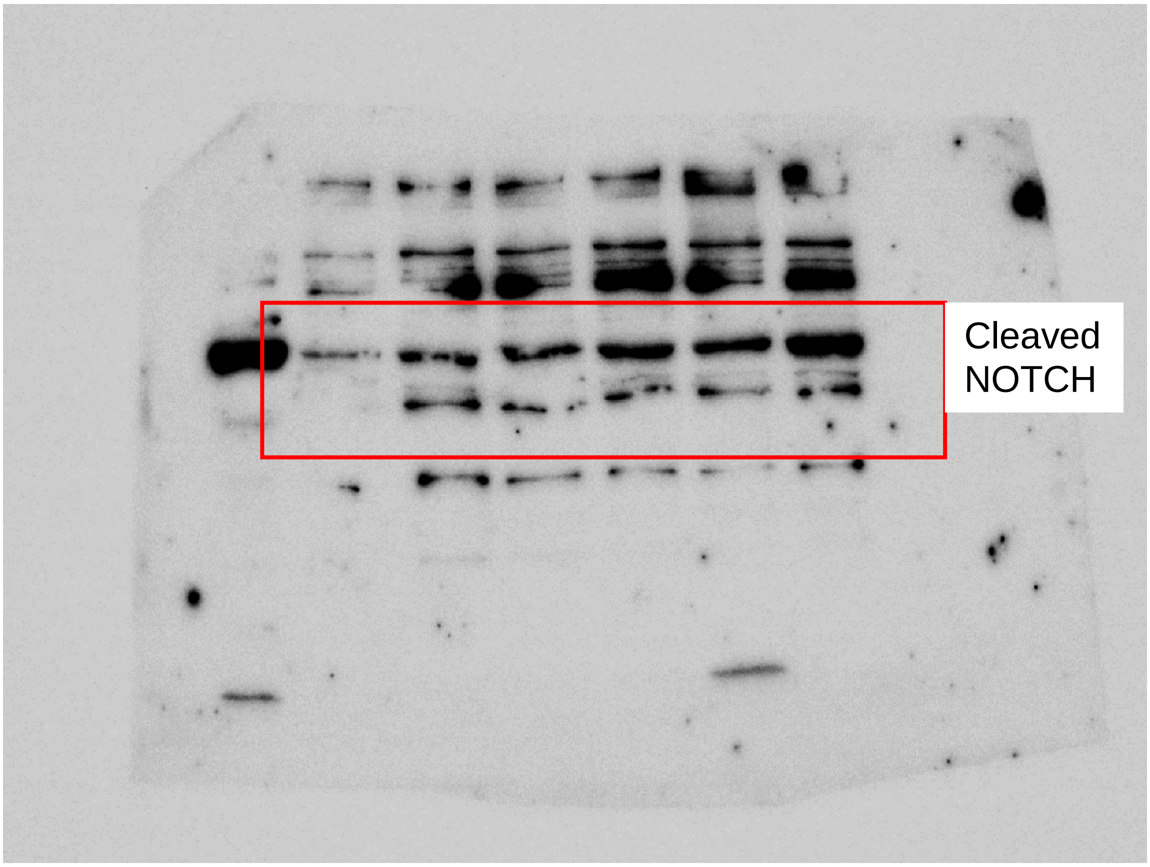

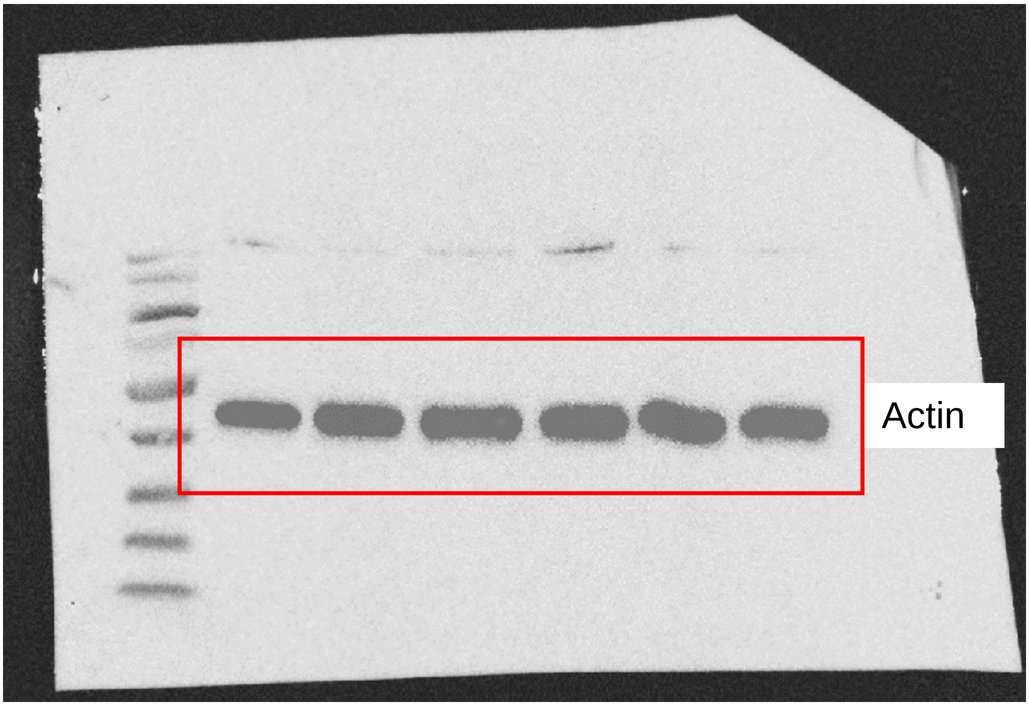

Supplemental Figure 6J

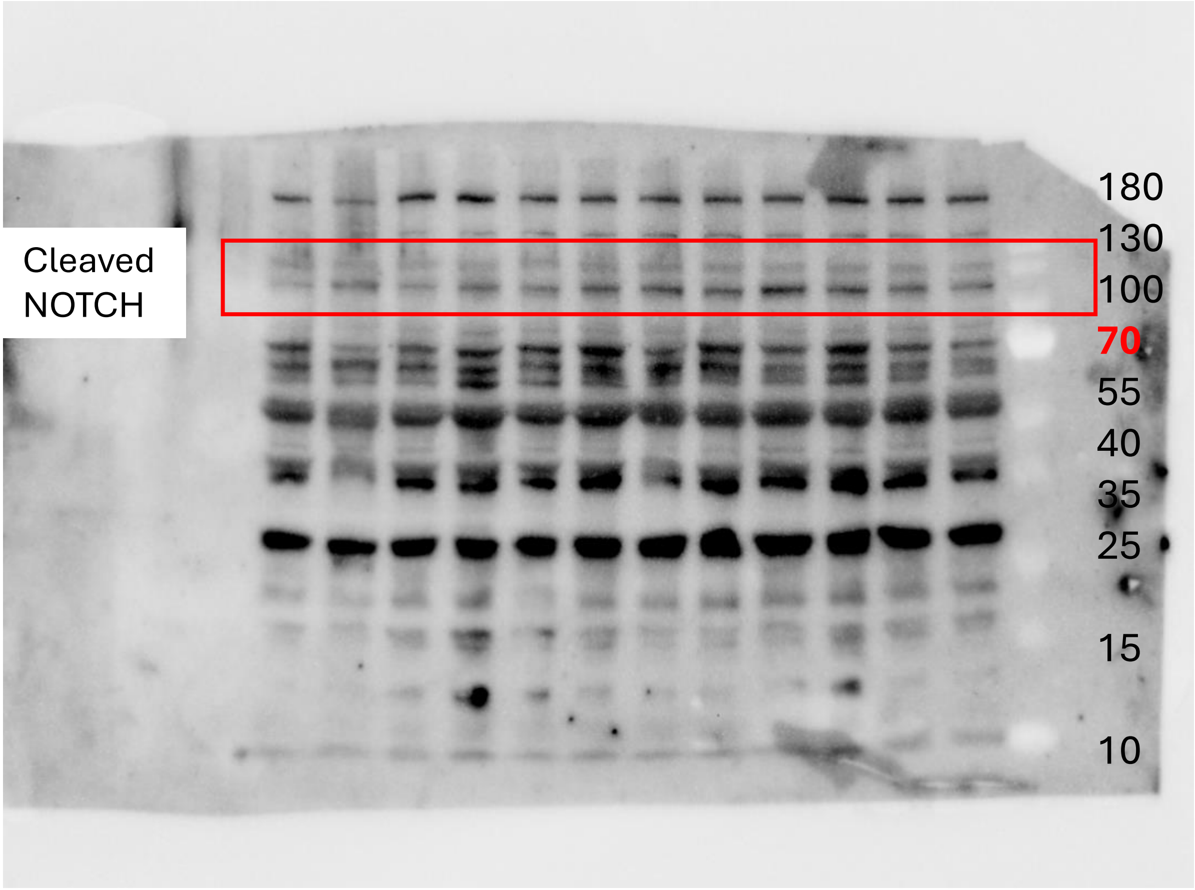

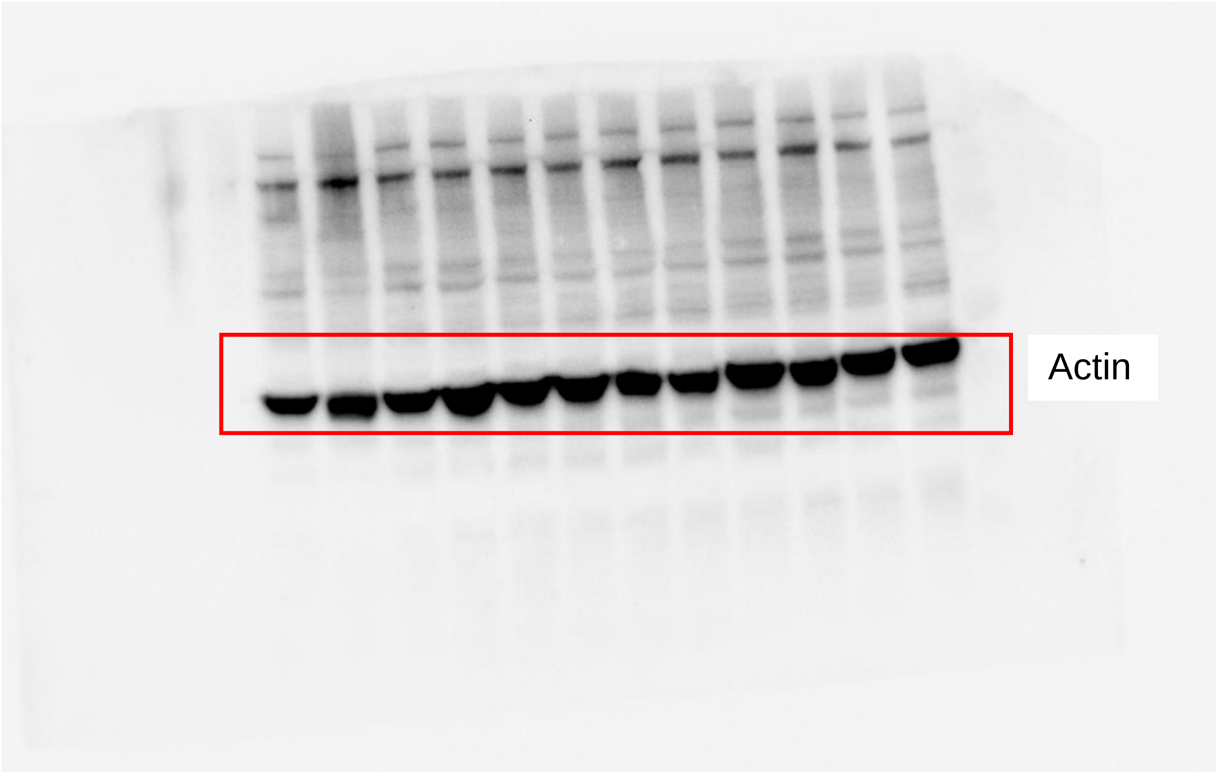

Supplemental Figure 6L

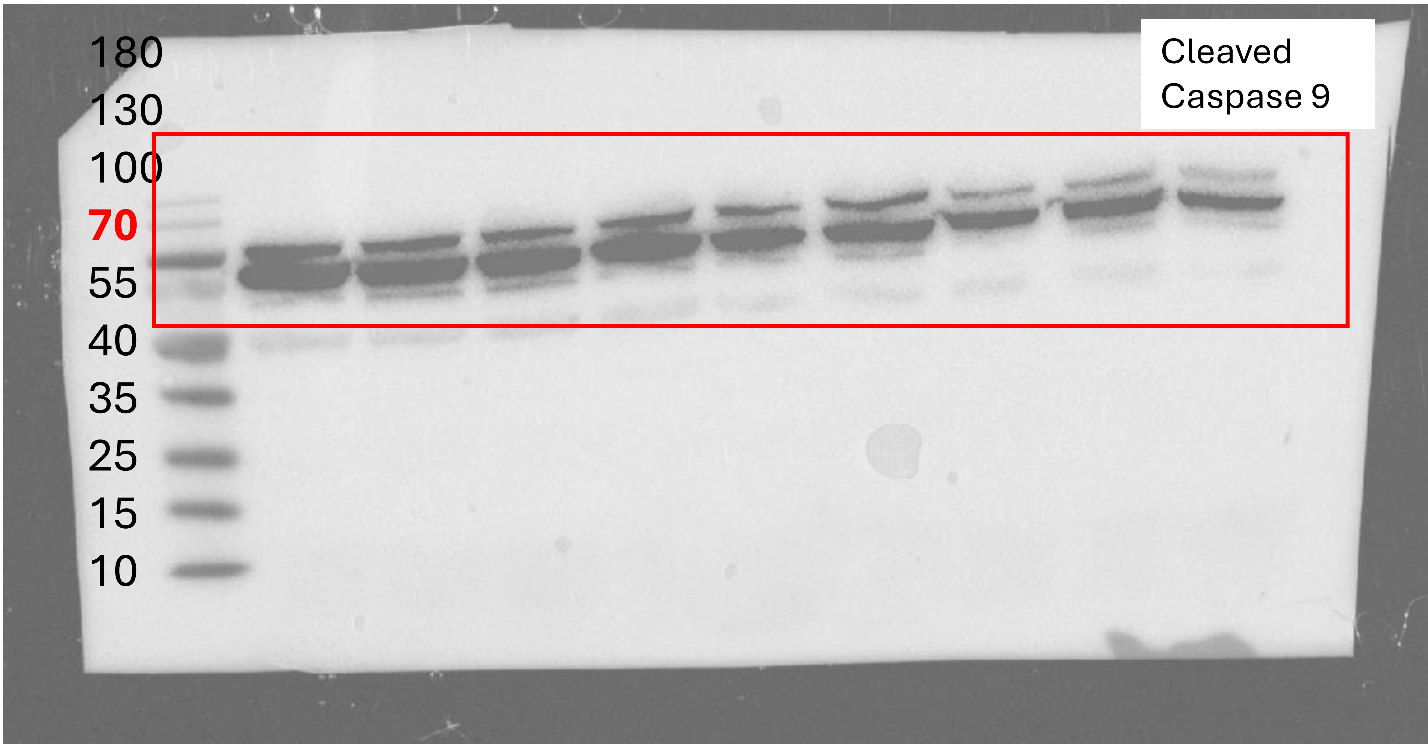

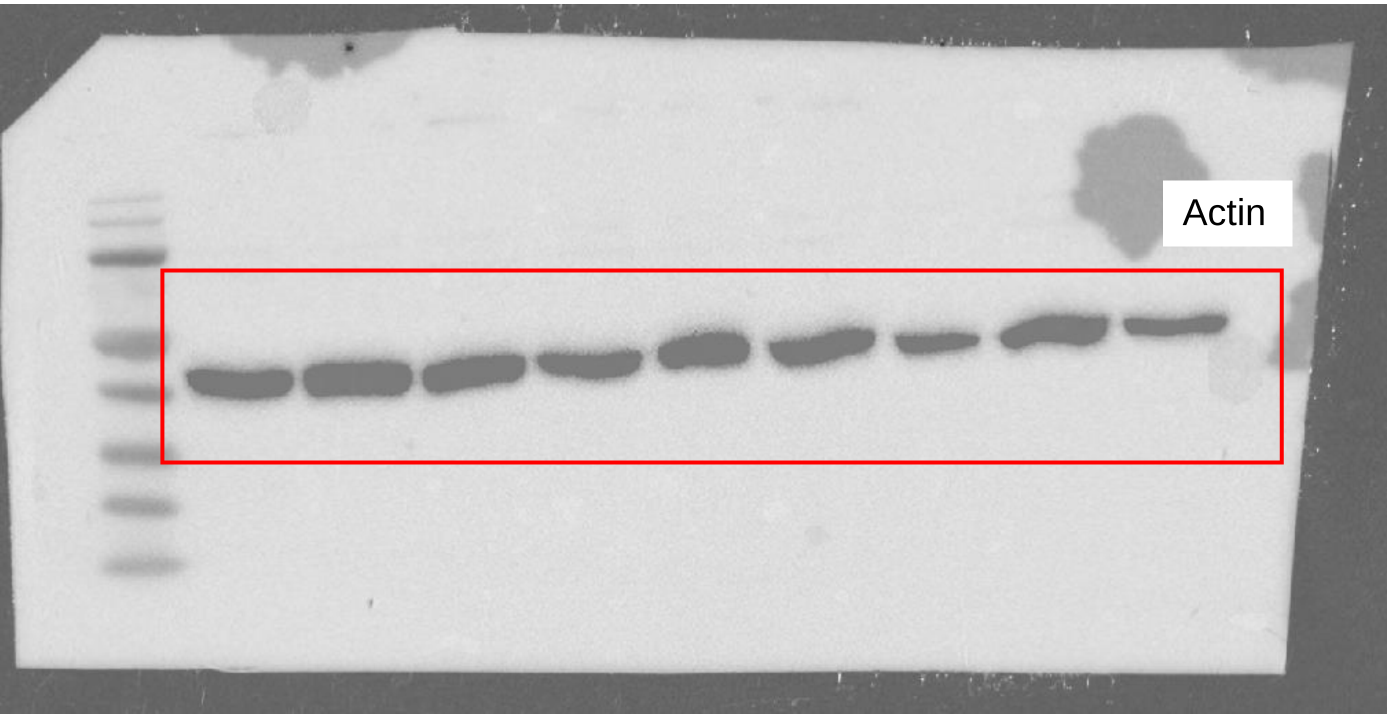

Supplemental Figure 8F

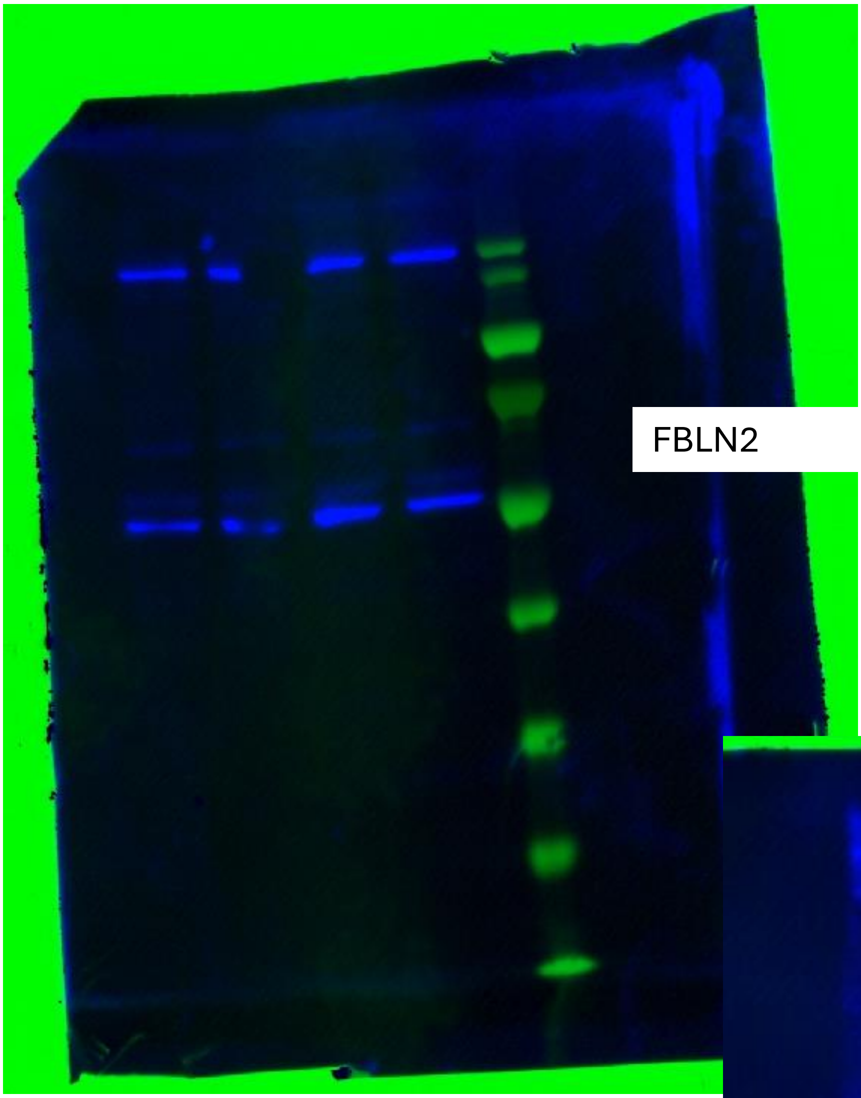

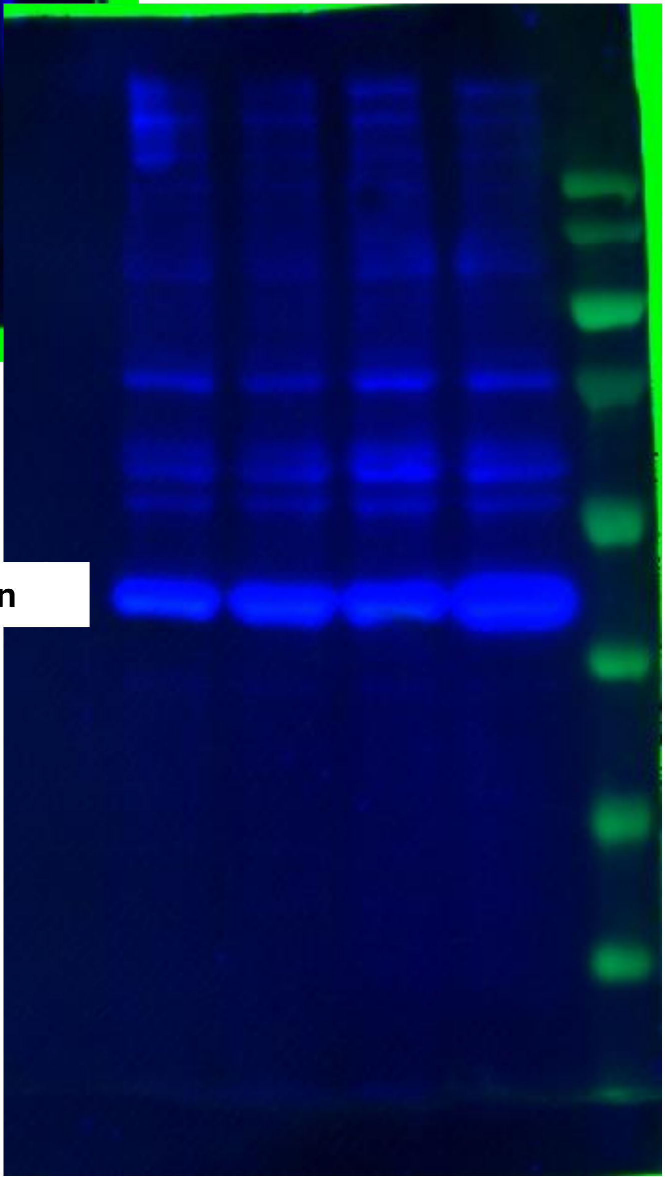

Supplemental Figure 8H

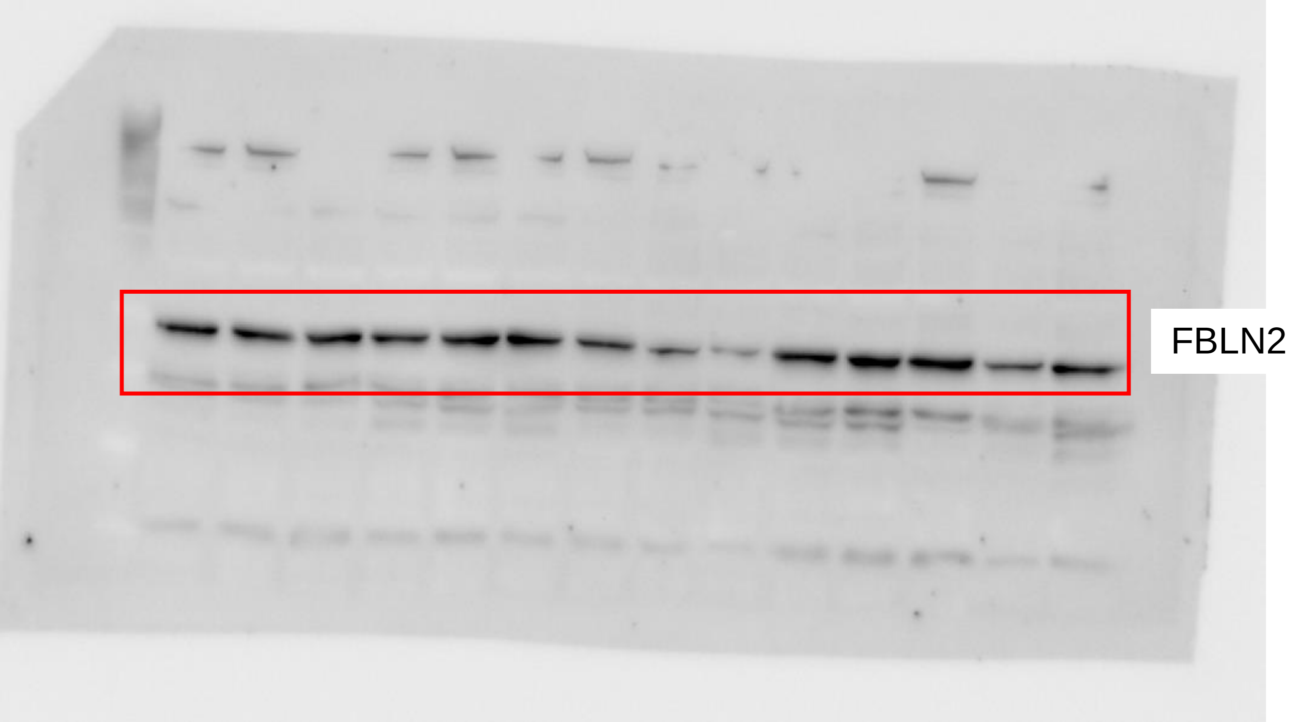

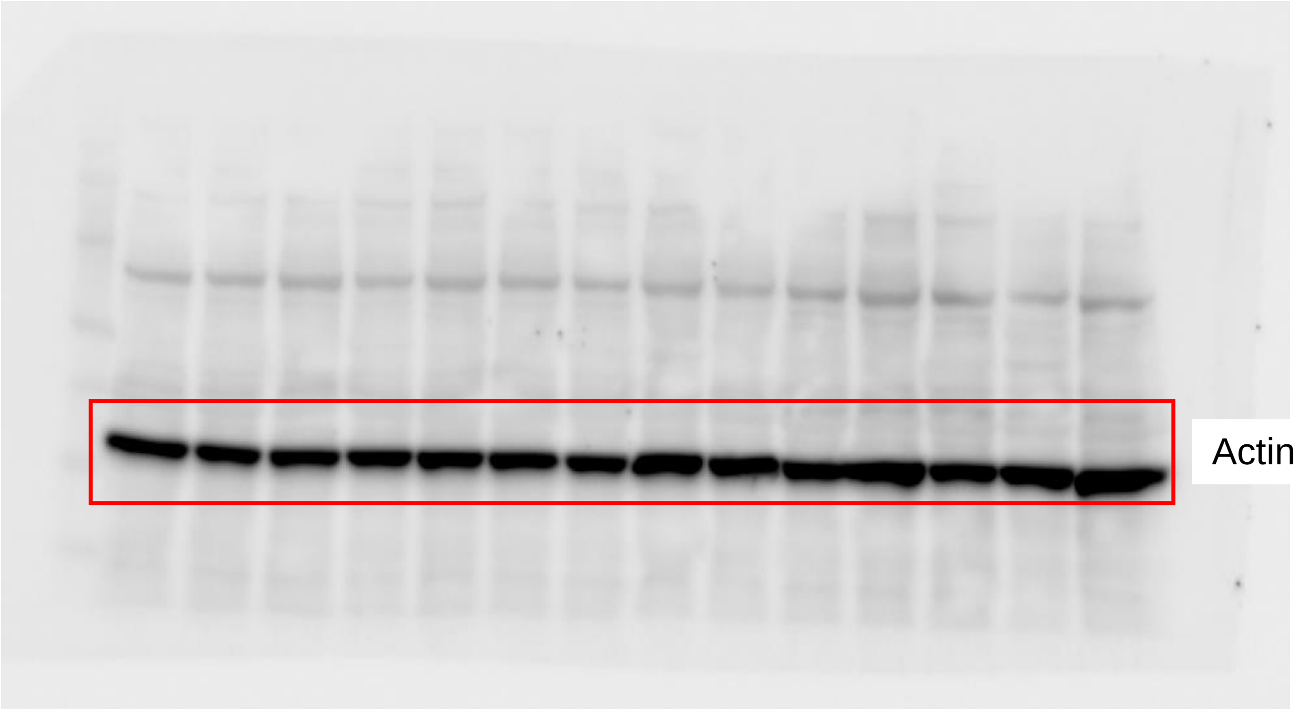

Supplement: Unedited blot and gel images [file jci-134-176910-s080.pdf]
